# Analytical Dual Flip Angle R1 Calculation Outside the Small‐Angle Regime

**DOI:** 10.1002/mrm.70174

**Published:** 2025-11-19

**Authors:** Luke J. Edwards, Kerrin J. Pine, Ilona Lipp, EBC consortium, Evgeniya Kirilina, Gunther Helms, Nikolaus Weiskopf, Catherine Crockford, Luke J. Edwards, Angela D. Friederici, Tobias Gräßle, Philipp Gunz, Carsten Jäger, Evgeniya Kirilina, Ilona Lipp, Kerrin Pine, Matyas Liptovszky, Nikolaus Weiskopf, Roman M. Wittig

**Affiliations:** ^1^ Department of Neurophysics Max Planck Institute for Human Cognitive and Brain Sciences Leipzig Germany; ^2^ Department of Cognitive Neuroscience, Faculty of Psychology and Neuroscience Maastricht University Maastricht the Netherlands; ^3^ Medical Radiation Physics, Clinical Sciences Lund Lund University Lund Sweden; ^4^ Felix Bloch Institute for Solid State Physics, Faculty of Physics and Earth System Sciences Leipzig University Leipzig Germany; ^5^ Wellcome Centre for Human Neuroimaging, Institute of Neurology University College London London UK

**Keywords:** ex vivo imaging, in vivo histology, longitudinal relaxation, T1 relaxometry

## Abstract

**Purpose:**

To evaluate a new analytical estimator for R1 and apparent proton density (A) from short‐TR dual flip angle data which does not rely on the small flip angle approximation and can thus be applied to a broader range of data, especially where relatively large flip angles are needed to achieve sufficient T1‐weighting for R1 estimation.

**Theory and Methods:**

A rational approximation of the Ernst equation was derived for small R1·TR and rearranged to give analytical estimators of R1 and A from dual flip angle data. Unlike previously used analytical estimators, this method relies neither on the flip angles being small nor the two TRs being equal or integer multiples of each other. R1 and A estimated using the novel method were compared to estimates using the conventional small‐angle approximation approach in simulations and data measured at 7T from six in vivo human participants and a postmortem chimpanzee brain. Test–retest scans of the six participants were used to evaluate within‐participant coefficients of variance (WCV) of R1 and A using the two methods.

**Results:**

The small‐angle approximation gave rise to a flip angle‐dependent bias in all cases. This bias was not observed using the novel method, demonstrating its higher accuracy. There were only negligible differences in WCV between the methods, demonstrating that precision is preserved.

**Conclusion:**

The increase in accuracy and preservation of precision suggest that the novel method should be used instead of the current small flip angle method.

## 1 Introduction

Dual flip angle R1 mapping is one of the workhorses of quantitative brain imaging^1^ [4, 5, 6]. It gives maps of R1 and a signal amplitude A proportional to proton density (PD) from two FLASH volumes recorded with different excitation flip angles ($\alpha_{1}$ and $\alpha_{2}$) and (potentially) different short (≪1/R1) repetition times (${\text{TR}}_{1}$ and ${\text{TR}}_{2}$) such that one is PD‐weighted (small flip angle and/or long 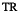) and the other T1‐weighted (large flip angle and/or short 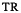). An efficient, robust and commonly used method for computing R1 and A from the two volumes uses a rational approximation of the Ernst equation derived under the assumption of small 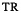
s and small flip angles [6, 7, 8, 9].

Dual flip angle mapping protocols covering just a few echoes at moderately high resolution (e.g., ∼1mm) allow 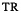 to be short enough that both PD‐weighting and T1‐weighting can be achieved with small flip angles, justifying use of the small‐angle approximation. However, acquiring enough echoes to also allow accurate R2* mapping [6, 7] or going to higher resolution necessitates extending 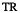, meaning higher flip angles are needed to obtain sufficient T1‐weighting for precise estimation of R1 and A, giving rise to R1 and A map biases when the standard rational approximation is used.

This bias becomes visually apparent in two important cases. First, large transmit B1 inhomogeneities over the brain at 7T [10] mean the flip angles can become large, for example, a nominal flip angle of $20^{\circ }$ can lead to actual flip angles above $30^{\circ }$ in some regions. Second, postmortem experiments often use even longer 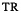
s than in vivo (trading acquisition time for higher resolution) and fixed tissue (with higher R1 due to fixation [11, 12]) meaning that the optimal flip angles can become very large.

Closed‐form estimators for R1 and A exist if ${\text{TR}}_{1}$ and ${\text{TR}}_{2}$ are equal [13, 14] or integer multiples of each other [15]. However, restricting the choice of the 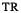
s may not be optimal for a given application; for instance Weiskopf et al. [16] chose the minimum 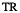 for one of the volumes to minimise acquisition time, but chose the 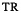 of the second volume to be equal to that of a contrast with additional magnetization transfer (MT)‐weighting to facilitate computation of the MT ratio (MTR). The choice of equal or integer‐multiple 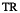
s may also be nonoptimal for estimating A, as we show in Section S1 in the Supporting Information. In general one could non‐linearly fit R1 and A to the steady state equations directly [17], but this will usually be slower than using a closed form solution. Efficient computation of R1 and A becomes particularly important for high‐resolution protocols, as the number of voxels scales cubically with the isotropic resolution.

Here we present and validate closed‐form approximate estimators of R1 and A from short‐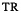 dual flip angle data which are accurate even when one (or both) of $\alpha_{1}$ and $\alpha_{2}$ are large.^2^

## 2 Theory

The Ernst equation giving the steady state signal $S_{n}$ under the assumption of perfect spoiling of transverse magnetization at the end of each 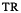 ^3^ for excitation flip angle $\alpha_{n}$ and repetition time ${\text{TR}}_{n}$ is [19]

(1) $$S_{n}=A\;\sin(\alpha_{n})\frac{1-\exp(-\text{R1}\;{\text{TR}}_{n})}{1-\cos(\alpha_{n})\exp(-\text{R1}\;{\text{TR}}_{n})}.$$

Herein $\alpha_{n}=f_{t}{\alpha_{n}}^{\text{(nominal)}}$ is the actual flip angle, where $f_{t}$ is the spatially varying ratio of actual and nominal flip angles and ${\alpha_{n}}^{\text{(nominal)}}$ is the nominal flip angle. It is convenient^4^ to make the half‐angle tangent substitutions $\sin(\alpha_{n})=\tau_{n}/(1+{\left(\tau_{n}/2\right)}^{2})$ and $\cos(\alpha_{n})=(1-{\left(\tau_{n}/2\right)}^{2})/(1+{\left(\tau_{n}/2\right)}^{2})$ where $\tau_{n}=2\tan(\alpha_{n}/2)$ so that [13]

(2) $$S_{n}=A\;\tau_{n}\frac{1-\exp(-\text{R1}\;{\text{TR}}_{n})}{1-\exp(-\text{R1}\;{\text{TR}}_{n})+{\left(\tau_{n}/2\right)}^{2}(1+\exp(-\text{R1}\;{\text{TR}}_{n}))}.$$

We use a Padé approximant [20, 21] to approximate this equation as it allows us to retain its rational form and gives a better representation of the function than the Taylor series.^5^ The [1/1] Padé approximant of Equation 2 around $\text{R1}\;{\text{TR}}_{n}=0$ is^6^

(3) $$S_{n}\approx A\;\tau_{n}\frac{\text{R1}\;{\text{TR}}_{n}}{{\tau_{n}}^{2}/2+\text{R1}\;{\text{TR}}_{n}}.$$

Equation 3 has the same form as the short‐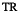 small flip angle signal results with $\tau_{n}$ instead of α [6, 7, 8]. We can therefore directly infer the analytic solutions for the estimation of R1 and A from two measurements $S_{1}$ and $S_{2}$ (cf. Equations (A.6) and (A.7) in Tabelow et al. [6]):

(4) $$\text{R1}\approx \frac{1}{2}\frac{\left(S_{1}\tau_{1}/{\text{TR}}_{1})-(S_{2}\tau_{2}/{\text{TR}}_{2}\right)}{\left(S_{2}/\tau_{2})-(S_{1}/\tau_{1}\right)}$$

and

(5) $$A\approx S_{1}S_{2}\frac{\left({\text{TR}}_{1}\;\tau_{2}/\tau_{1})-({\text{TR}}_{2}\;\tau_{1}/\tau_{2}\right)}{S_{2}{\text{TR}}_{1}\;\tau_{2}-S_{1}{\text{TR}}_{2}\;\tau_{1}}.$$

## 3 Methods

### 3.1 Simulations

We simulated experimental in vivo and postmortem protocols using Equation 1, estimated R1 and A using the small‐angle approximation estimators [6] and the estimators from Equations (4) and (5), and compared the estimates to the simulation ground truth. A was arbitrarily set to 1 in both cases.

The in vivo simulation used $\text{R1}=0.82\;s^{-1}$, typical of human brain white matter (WM) at 7T [22], the same 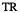
s and flip angles as the in vivo study, and $f_{t}$ ranging from 45% to 135% of the nominal flip angle, matching the range over WM in vivo.

The postmortem simulation used $\text{R1}=2\;s^{-1}$ [23], the same 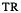
s and flip angles as the postmortem study, and $f_{t}$ ranging from 60% to 110%, matching the range over the postmortem sample.

### 3.2 In Vivo Measurements

Six healthy human participants (22–28y, mean 25.8y; 4f, 2m) were each scanned twice using an ultrahigh resolution multiparametric mapping (MPM) protocol [16, 24, 25] on a 7T Magnetom MRI scanner (Siemens Healthcare, Erlangen, Germany) with a 1‐channel transmit/32‐channel receive RF head coil (Nova Medical, Wilmington, MA, USA) after giving informed consent. The two scanning sessions were on consecutive days in all cases except one, where they were four days apart. The study was conducted under the approval of the Ethics Commission at the Medical Faculty of Leipzig University.

In each session, we acquired two gradient and RF spoiled multi‐echo 3D gradient echo FLASH datasets^7^ (400μm isotropic resolution, ${\text{TR}}_{1}={\text{TR}}_{2}=31.6\;\text{ms}$, 8 equispaced TE from 3.3 to 21.5 ms, bandwidth: 434 Hz/pixel, matrix size: 560/640/416 (phase1/read/phase2), partially parallel imaging factor [26] 2 in each phase‐encoding direction, integrated k‐space reference lines: 88/84 (phase1/phase2), ${\alpha_{1}}^{\text{(nominal)}}=5^{\circ }$, ${\alpha_{2}}^{\text{(nominal)}}=27^{\circ }$, 6π per pixel gradient spoiling at $26\;\text{mT}m^{-1}$ gradient strength per 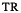, RF spoiling increment $137^{\circ }$, acquisition time (TA): 32 min for each dataset, participant motion monitored and corrected for using a prospective motion correction (PMC) system (Kineticor, Honolulu, HI, USA) [27, 28], k‐space data retrospectively reconstructed using a SENSE‐based algorithm [29, 30, 31] implemented in MATLAB (The MathWorks, Natick, MA, USA)), and 3D‐EPI spin echo–stimulated echo (SE/STE) $f_{t}$ mapping data for flip angle correction [10] (4 mm isotropic resolution, 15 equispaced spin echo flip angles from $120^{\circ }$ to $330^{\circ }$, mixing time: 34.91 ms, TA: 6 min including a B0 map for EPI distortion correction). The PMC system's reference position locking system maintained within‐session positioning between FLASH datasets. Two dielectric pads (Multiwave Imaging SAS, Marseille, France) [32], placed around each participant's head (one each side) at approximately the level of the temporal lobe, reduced $f_{t}$ inhomogeneity. The transmit voltage was calibrated so that the nominal flip angle was achieved over the occipital lobe using a low‐resolution transmit field map (Siemens WIP658; mean±standard deviation voltage over all participants and sessions 243V±18V).

We calculated R1 and A for each session using the hMRI toolbox [6], setting the parameter hmri_def.small_angle_approx in the toolbox configuration file to true to use the small‐angle approximation and false to use the novel method. We disabled registration between the FLASH datasets as they were already well aligned by PMC‐based position locking, and disabled A receive bias correction and calibration [6] to avoid it confounding the analysis.

R1 and A were also computed using exact analytical solutions [23] (which exist as the two 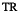
s are equal) [13] using the R2*‐corrected PD‐ and T1‐weighted volumes and $f_{t}$ map generated by the hMRI toolbox.

### 3.3 In Vivo Analysis

The R2*‐corrected PD‐ and T1‐weighted volumes generated by the hMRI toolbox [6] were jointly segmented using unified segmentation [33] in SPM12 (https://github.com/spm/spm12) with improved tissue priors [34], giving probability maps of grey matter (GM), WM and cerebrospinal fluid (CSF).

A WM mask was generated for each participant and session by thresholding each WM probability map at 99%. This threshold was based on visual inspection of the generated masks. The $f_{t}$ map generated by the hMRI toolbox was linearly interpolated to MPM space. Relative errors were computed as percent differences of R1 and A estimated using the small angle and novel methods to estimates using the exact analytical solution, and binned based on $f_{t}$. The median and 95% confidence intervals of these bins were then plotted as histograms against $f_{t}$. The plotted range of $f_{t}$ covered the 99% confidence intervals of $f_{t}$ over the WM masks of all participants and sessions (45% to 135% rounded to 5%) to avoid analysing bins with only a small number of samples.

Next, we visualised the spatial distribution of the bias from using the small‐angle approximation, treating the novel method results as silver standard. A brain mask for visualisation was generated by taking all voxels where the probability of GM, WM or CSF was greater than zero and then dilating and eroding to fill holes in the mask. Relative errors within the WM mask were also plotted as 2D density histograms as a function of $f_{t}$.

Between‐session test–retest reliability was evaluated within the WM mask under the assumption that WM is mostly homogenous. Only voxels with $f_{t}$ within the 99% confidence intervals were included for consistency with the histograms and to avoid $f_{t}$ outliers. The mean of each parameter within this mask was used to calculate the within‐participant coefficient of variation (WCV) [35] for each method, a reproducibility measure quantifying the inconsistency between test and retest.

### 3.4 Postmortem Measurements and Analysis

We also tested the method on a postmortem dataset from Lipp et al. [23] of a chimpanzee (43y; f) scanned on a 7T Terra MRI scanner (Siemens Healthcare, Erlangen, Germany) with a 1‐channel transmit/32‐channel RF head coil (Nova Medical, Wilmington, MA, USA). As in the in vivo MPM protocol, we acquired two FLASH datasets (300μm isotropic resolution, ${\text{TR}}_{1}={\text{TR}}_{2}=70\;\text{ms}$, 12 equispaced TE from 3.63 to 41.7 ms, bandwidth: 331Hz/pixel, matrix size: 432/378/288, ${\alpha_{1}}^{\text{(nominal)}}=18^{\circ }$, ${\alpha_{2}}^{\text{(nominal)}}=84^{\circ }$, 6π per pixel gradient spoiling at $37.8\;\text{mT}\;m^{-1}$ gradient strength per 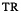, RF spoiling increment $137^{\circ }$, scanner reconstruction), and $f_{t}$ mapping data for flip angle correction.

Again, we computed R1 and A using the small‐angle approximation, novel method, and exact analytical solutions, without A receive bias correction and calibration. Although the brain did not move during the scanning session, we registered the FLASH datasets to correct apparent motion due to scanner drift.

Results were analyzed within a brain mask derived from thresholding the first T1‐weighted volume [23], and the $f_{t}$ map was linearly interpolated to MPM space. Median and 95% confidence intervals of the relative errors of the parameter estimates to the exact solution within the brain mask were plotted as histograms against $f_{t}$, and bias in the spatial distribution from using the small‐angle approximation was visualised using the novel method result as silver standard. The plotted range of $f_{t}$ again covered the 99% confidence intervals of $f_{t}$ over the brain mask (60% to 110% rounded to 5%).

## 4 Results

**FIGURE 1:**
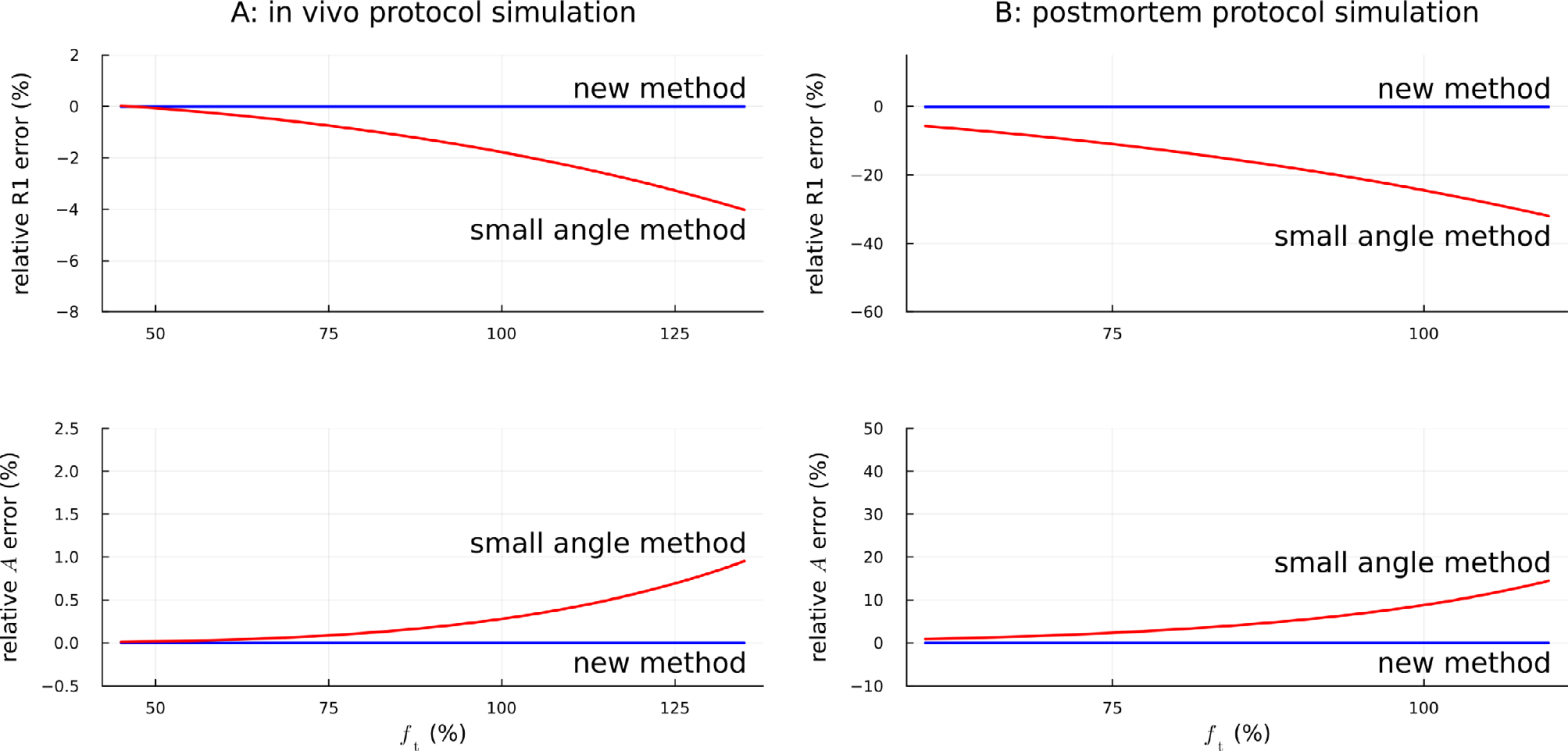
Simulations showing the bias induced in R1 and A for different values of $f_{t}$ when using the small‐angle approximation (red lines). The novel estimators (Equations 4 and 5) remained accurate over the whole range (blue lines). All results here and in the following were computed using the local flip angles. A: The error in R1 reached 4% for large values of $f_{t}$ in the in vivo protocol. B: The much larger flip angles in the postmortem protocol meant that the error in R1 and A became very large. Note that A and B have different axis scales for relative error and $f_{t}$.

Figure 1 shows the benefits of the novel approximation for simulated in vivo and postmortem 7T protocols. The benefit was particularly large for the postmortem protocol due to the large flip angles.

**FIGURE 2:**
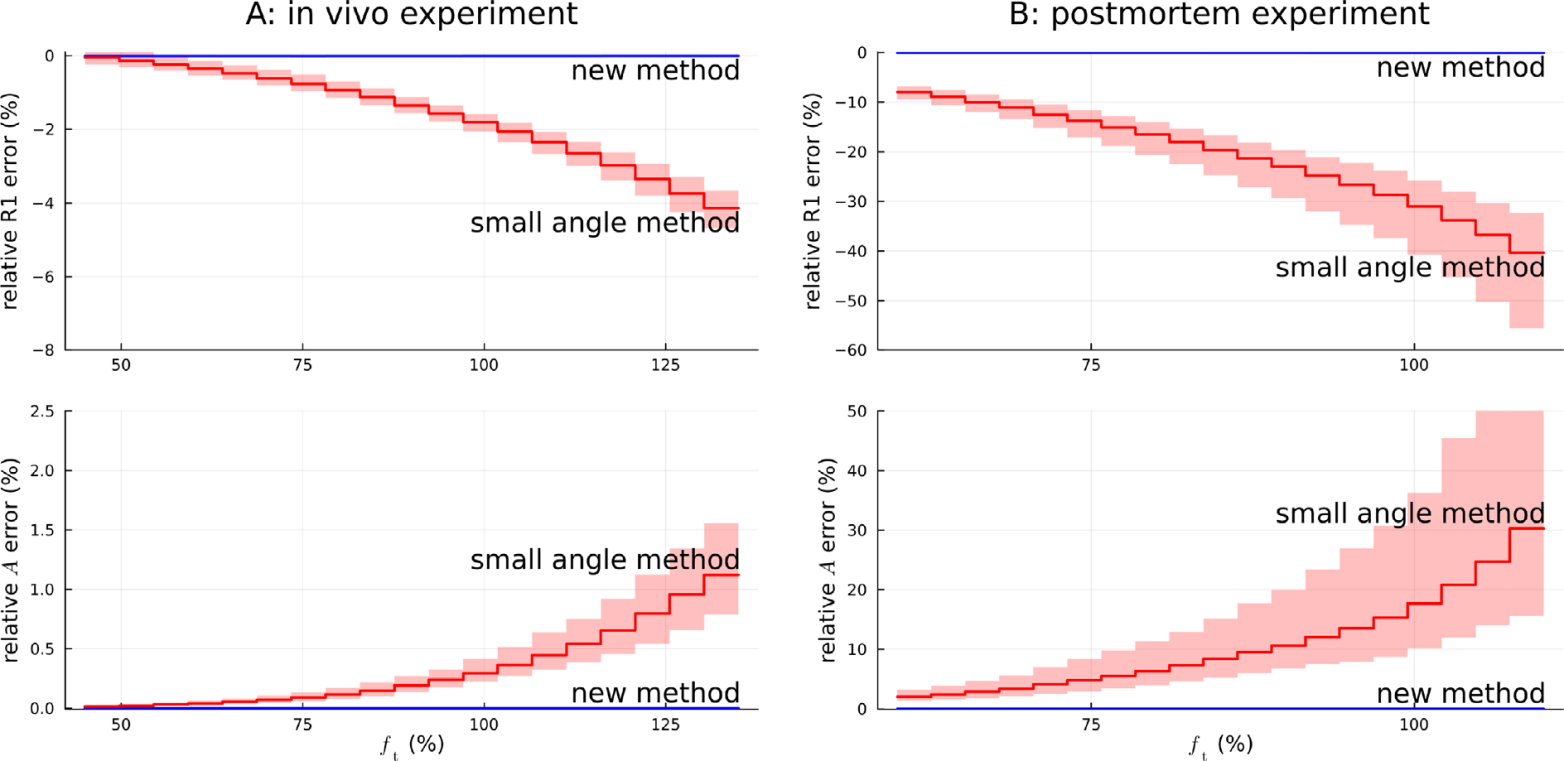
Experimental histograms show that using the small‐angle approximation biased R1 and A for larger $f_{t}$ values (red lines), whereas the novel method remained accurate over the whole range (blue lines), relative to results using exact analytical solutions. Solid lines give medians within the bins, shaded areas 95% confidence intervals. The results accorded well with the predictions from the simulations in Figure 1. *Note*: A and B have different axis scales for the errors. A: in vivo over WM mask (participant 1, session 1). B: postmortem over brain mask.

Figure 2 shows the benefits of the novel approximation for experimental in vivo and postmortem 7T data. The bias from using the small‐angle approximation was comparable to the simulations in Figure 1 (red solid lines). There was also a visible increase in the width of the 95% confidence intervals of the relative parameter differences with increasing $f_{t}$ when using the small‐angle approximation (red shaded regions). The novel estimators remained accurate over the whole range of $f_{t}$ (blue solid lines; confidence intervals not visible at this line width).

**FIGURE 3:**
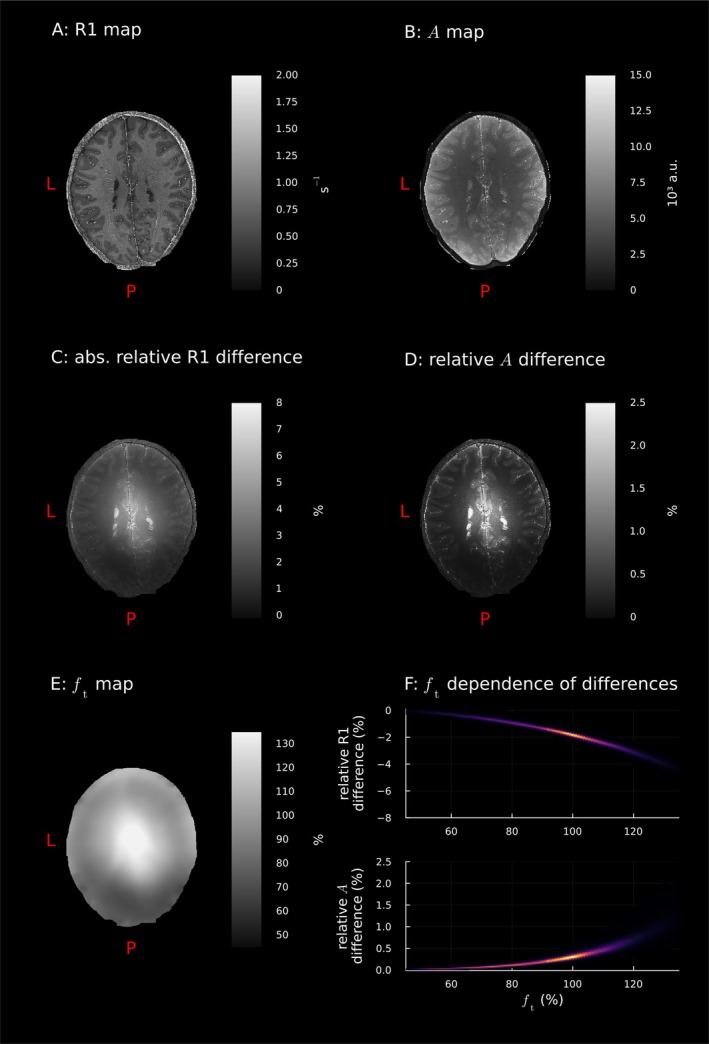
The spatial distribution of differences between small angle and novel estimator results followed the spatial distribution of the $f_{t}$ map in an in vivo participant (participant 1, session 1). A: R1 and B: A maps estimated using the novel estimators in an exemplary slice. C and D: Relative differences of small angle and novel estimator results show the same spatial pattern as E: the $f_{t}$ map (interpolated to MPM space). F: Histograms of the $f_{t}$‐dependence of the errors over WM (brighter colour means more voxels in a bin). Data from all participants can be found in Supporting Information: Figures S5–S16. abs.: absolute value; a.u.: arbitrary units; L: left; P: posterior.

**FIGURE 4:**
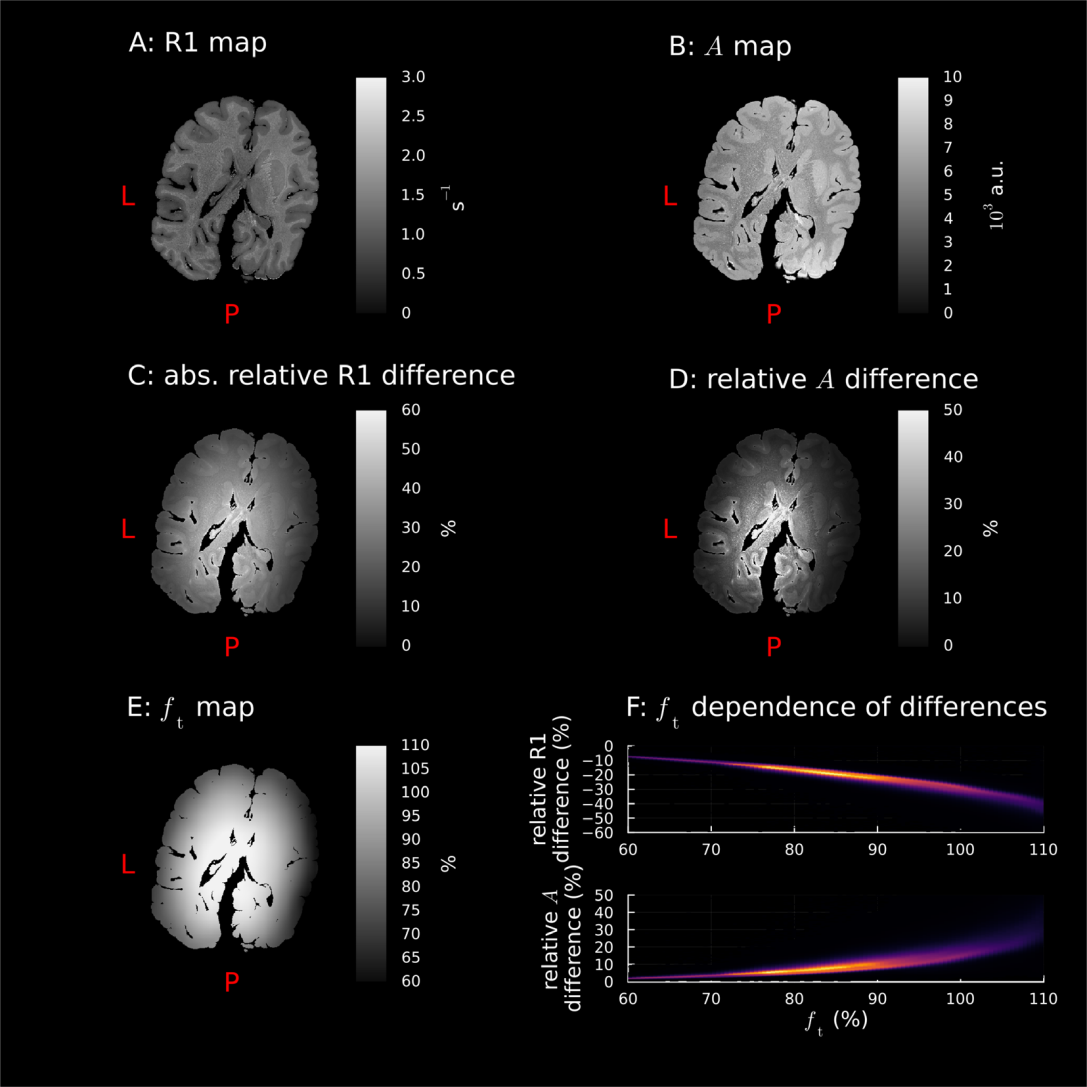
The spatial distribution of differences between small angle and novel estimator results followed the spatial distribution of the $f_{t}$ map in the postmortem chimpanzee brain. A: R1 and B: A maps estimated using the novel estimators in an exemplary slice. C and D: Relative differences of the small‐angle estimates to the novel estimator results show the same spatial pattern as E: the $f_{t}$ map (interpolated to MPM space). F: Histograms of the $f_{t}$‐dependence of the errors over the brain (brighter colour means more voxels in a bin). The brain is slightly rotated in plane as it was not perfectly aligned to the acquired field of view. abs.: absolute value; a.u.: arbitrary units; L: left; P: posterior.

Figure 3 shows that the spatial distribution of the differences between the two approximations followed the spatial distribution of the $f_{t}$ map. This bias was reproducible across participants and sessions, as shown by the similarity of the results of the other datasets in Section S3 in the Supporting Information. This pattern could also be seen in the postmortem dataset (Figure 4) with even larger relative errors due to the larger flip angles.

The reproducibility metric WCV computed from the in vivo data was slightly smaller for the novel estimator and exact solution than the small‐angle approximation in R1 and slightly larger in A (Table 1), although the differences appear negligible. For R1, the WCV was smaller than the largest bias from using the small‐angle approximation, and for A it was comparable (Figure 2).

**TABLE 1:** Within participant coefficient of variation (WCV) of MPM parameters with different approximations for the in vivo data. Smaller is better.

MPM	Approximation	WCV (%)
A	exact	1.708
	Padé approximant	1.708
	small angle	1.702
R1	exact	1.370
	Padé approximant	1.367
	small angle	1.407

## 5 Discussion

By using a Padé approximant in 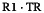 of the Ernst equation after half‐angle tangent substitution we derived an analytic solution to estimate R1 and A from dual flip angle R1 mapping protocols. Compared to a commonly used estimation method using the small‐angle approximation, our method was more accurate, removing bias in both in vivo and postmortem acquisitions in line with simulations.

The new method preserved the reproducibility (as measured by WCV) of the MPM parameters in an in vivo experiment compared to the previously extensively validated approach using the small‐ angle approximation [9]. However, one may expect that removing bias would lead to an increase in reproducibility. This discrepancy can be explained by two factors.

First, the bias would only show up as a reproducibility decrease if the bias differs between the two repetitions. As the spatial variation of the bias is related to the spatial variation of $f_{t}$, this means that reproducibility is related to how much the $f_{t}$ map differs between the two repetitions. The $f_{t}$ map is generally relatively slowly varying over most of the brain and a function of both the shape of the head and the position of the head in the transmit coil. As the same participant was placed in approximately the same position and the $f_{t}$ adjustment procedures were identical, $f_{t}$ differences will be limited between the acquisitions, leading to cancellation of most of the between‐session $f_{t}$‐bias.

Second, the A map in particular is affected by both the transmit B1 field and the receive B1 field [5, 6]. We correct for transmit‐related bias by avoiding the small‐angle approximation, but receive bias will remain. The geometry of multi‐channel receive coils, which have highest sensitivity at the edge of the brain [36], mean the receive bias spatial distribution is similar to the inverse of the transmit bias (cf. the spatial distribution of $f_{t}$ in Figures 3 and 4). The slightly larger WCV when using the small‐angle approximation could be due to the receive bias partially compensating for the bias in the A estimates due to the small‐angle approximation.

Nevertheless, the WCV was in all cases less than (R1) or comparable to (A) the largest bias from using the small‐angle approximation in the in vivo experiment. This emphasises the importance of accounting for this bias to obtain accurate maps.

We retained the assumption that 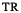 is short relative to R1. Exact solutions without approximation are possible when ${\text{TR}}_{1}=n{\text{TR}}_{2}$ for integer n [13, 14, 15]; however, we wished to retain the flexibility to choose ${\text{TR}}_{1}$ and ${\text{TR}}_{2}$ independently to provide greater freedom during imaging protocol optimisation. Non‐linear fitting could estimate R1 and A from Equation (2) directly for non‐equal 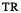
s [17], but will be much slower than using the closed form solution presented here. Higher‐order Padé approximants might give higher accuracy, but were not considered here as they lead to higher order polynomials with nonunique solutions.

The approach derived here can be extended to fit R1 and A from more than two FLASH volumes by linearising Equation (3) or (S1) in a DESPOT1‐like manner [37]. This allows DESPOT1 fitting in cases where 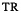 is not equal between the volumes, though it adds the assumption that 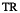 must be short relative to R1.^8^

For simplicity, we omitted corrections for imperfect spoiling, MT, and myelin water contributions in our analysis, which also increase with increasing flip angle [8, 38, 39, 40, 41]. Combination of the proposed method with imperfect spoiling correction of R1 is available in the hMRI toolbox, however, bias due to imperfect spoiling was much smaller than bias due to the small‐angle approximation for our protocols (see Section S4 in the Supporting Information). Correction for MT and myelin water could be incorporated by modifying the excitation pulses to balance MT and therefore myelin water contributions [38] or modifying the R2* fitting procedure to remove myelin water contributions [39, 41]. However, the default hMRI toolbox R2* fitting procedure used was previously found to be a good trade‐off between myelin water contributions and SNR [41].

Inhomogeneity in $f_{t}$ will also lead to the flip angles moving away from optimal values from the perspective of optimal noise propagation. This leads to an increase in variance in the maps which we did not consider above. Simulations in Section S5 in the Supporting Information show that the variance propagated to the small angle and novel method estimates are similar.

Parallel transmit (pTx) can be used to mitigate $f_{t}$ inhomogeneity [42, 43, 44]. This would reduce the spatial differences due to the use of the small‐angle approximation, but a constant bias would remain which is removed by using the novel method.

Inaccuracies in the $f_{t}$ map used for flip angle correction can also be a source of bias which we did not consider here [45], although measurements with two different $f_{t}$ mapping methods gave very similar results in a phantom (Section S6 in the Supporting Information).

The tested protocols each had equal 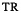 between the two weighted datasets, allowing use of an exact solution to provide reference A and R1 estimates. However, the novel method can also provide accurate results when the 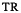
s are not equal, as demonstrated by comparison to inversion recovery R1 mapping in a phantom in Section S6 in the Supporting Information.

Calibration procedures to convert A into a proton density map [46] could remove most of the A bias, for example, by modeling the bias field as a sum of Gaussian functions [6, 33], though they will not capture all of the bias from using the small‐angle approximation.

## 6 Conclusions

R1 and A estimated using a commonly used method that assumes small excitation flip angles showed bias in vivo and postmortem protocols at 7T. This bias was eliminated using a novel Padé approximant‐based estimator without the small‐angle approximation. We recommend using this estimator in future to improve accuracy.

## Conflicts of Interest

The Max Planck Institute for Human Cognitive and Brain Sciences and Wellcome Centre for Human Neuroimaging have institutional research agreements with Siemens Healthcare. N.W. holds a patent on acquisition of MRI data during spoiler gradients (US 10,401,453 B2). N.W. was a speaker at an event organized by Siemens Healthcare and was reimbursed for the travel expenses.

## Supporting information




**Data S1.** Supporting Information.

## Data Availability

The data that support the findings of this study are available on request from the corresponding author. The data are not publicly available due to privacy or ethical restrictions. The method presented here has been incorporated into the open source hMRI toolbox and is available from v0.2.5 and later (hmri.info), allowing others to apply the method presented here to efficiently extract more accurate R1 estimates from their dual flip angle data. The SPM and hMRI toolbox scripts used to calculate and process the maps, as well as the julia scripts used to calculate the statistics and generate the figures can be found at https://github.com/lukeje/large‐angle‐paper‐scripts.

## ENDNOTES

^1^
Comparison with other R1‐mapping techniques is beyond the scope of this Technical Note. Discussion of the trade‐offs between different methods can be found in prior literature, for instance [1, 2, 3].

^2^
An earlier version of part of this work was presented at the 2021 ESMRMB online meeting [18].

^3^
While imperfect spoiling will impact our estimate of R1 and A, this bias can be removed post hoc [8]. An analysis of R1 estimates including imperfect spoiling correction can be found in Section S4 in the Supporting Information, where we find that in our protocols the bias due to imperfect spoiling is much smaller than the bias from using the small‐angle approximation.

^4^
Though not required; an approximation using Equation 1 directly can be found in Section S2 in the Supporting Information.

^5^
A Padé approximant of order [m,n] is the best rational approximation of a function f(x) as g(x)/h(x) where g(x) is a polynomial of order m and h(x) is a polynomial of order n such that the Taylor series expansions of f(x) and g(x)/h(x) match to order m+n [20, 21].

^6^
The [1,1] Padé approximant was calculated using pade from the MATLAB Symbolic Math Toolbox and then verified and simplified by hand.

^7^
The nominal flip angles were chosen to obtain optimal R1 SNR from the perspective of noise propagation; see Figure S1 in the Supporting Information.

^8^
An implementation can be found at https://github.com/lukeje/n‐contrast‐mpm.

## Appendix A EBC (Evolution of Brain Connectivity) Consortium

Catherine Crockford (Institute of Cognitive Sciences, CNRS, FR), Luke J. Edwards (Max Planck Institute for Human Cognitive and Brain Sciences, DE), Angela D. Friederici (Max Planck Institute for Human Cognitive and Brain Sciences, DE), Tobias Gräßle (Robert Koch‐Institute, DE), Philipp Gunz (Max Planck Institute for Evolutionary Anthropology, DE), Carsten Jäger (Max Planck Institute for Human Cognitive and Brain Sciences, DE), Evgeniya Kirilina (Max Planck Institute for Human Cognitive and Brain Sciences, DE), Ilona Lipp (Max Planck Institute for Human Cognitive and Brain Sciences, DE), Matyas Liptovszky (Twycross Zoo, UK), Kerrin Pine (Max Planck Institute for Human Cognitive and Brain Sciences, DE), Nikolaus Weiskopf (Max Planck Institute for Human Cognitive and Brain Sciences, DE), Roman M. Wittig (Institute of Cognitive Sciences, CNRS, FR).
