# Supplementary material for: Analytical Dual Flip Angle R1 Calculation Outside the Small‐Angle Regime
Source: Magn Reson Med. 2025 Nov 19;95(4):2420–8. doi: 10.1002/mrm.70174 (PMC12850634; doi:10.1002/mrm.70174)
Supplement: Supplementary file 1 — Data S1. Supporting Information. [file MRM-95-2420-s001.pdf]

Supplementary information for "Analytical dual flip angle R1 calculation outside the small angle regime"

Luke J. Edwards[1,2,*], Kerrin J. Pine[1], Ilona Lipp[1], EBC consortium, Evgeniya Kirilina[1], Gunther Helms[1,3], and Nikolaus Weiskopf[1,4,5]

[1]Department of Neurophysics, Max Planck Institute for Human Cognitive and Brain Sciences, Leipzig, Germany

[2]Department of Cognitive Neuroscience, Faculty of Psychology and Neuroscience, Maastricht University, Maastricht, Netherlands

[3]Medical Radiation Physics, Clinical Sciences Lund, Lund University, Lund, Sweden

[4]Felix Bloch Institute for Solid State Physics, Faculty of Physics and Earth System Sciences, Leipzig University, Germany

[5]Wellcome Centre for Human Neuroimaging, Institute of Neurology, University College London, London, UK

[*]Corresponding author. Address: Department of Cognitive Neuroscience, Faculty of Psychology and Neuroscience, Maastricht University, Oxfordlaan 55, Maastricht 6229 EV, NL. email: luke.edwards@maastrichtuniversity.nl

S1 Unequal TR between flip angles can be the optimal choice for A

Dathe et al. derived flip angle pairs which minimised the first order propagated error in either R1 or A given a fixed TR which is the same for both contrasts.[1] Allowing TR to vary between the two volumes, however, can allow for further optimisation in the A case. We demonstrate this in Figure S1, where linear combinations of the first order error in Equations (4) and (5) were minimised while constraining the sum of the TRs of the two volumes. For R1 the result reproduced the result of Dathe et al.,[1] but increasing the weighting of A caused the optimal TRs to become unequal.

Figure S1: Changing the weighting of which parameter's error (σ^2(parameter)) should be minimised changes both the optimal flip angles and the TRs. Each optimal TR value is labelled with its respective optimal flip angle. Lower flip angles and longer TRs will tend to give more PD-weighting, higher flip angles and shorter TRs more T1-weighting. We optimised using the first order error propagation results from the supplementary information of Mohammadi et al.[2] with each flip angle α_n replaced with τ_n. To avoid bias from the different magnitudes of A and R1, relative rather than absolute errors were used in the linear combinations of parameter errors. This was achieved by scaling the errors by the respective A and R1 values. The R1 used in the optimisation matched the typical in vivo white matter value at 7T used for the simulation in the main manuscript ($0.82\,\mathrm{s}^{-1}$),[3] and the sum of the TRs was constrained to be no greater than $2 \times 31.6\,\mathrm{ms}$ so that the theoretical total acquisition time for the two volumes would not be greater than the total acquisition time used for the in vivo protocol. The flip angles used in the in vivo study in the main manuscript were lower than the R1-optimised values here as a compromise to account for the lower R1 of cortical grey matter[3] and reduce specific absorption rate (SAR).[4]

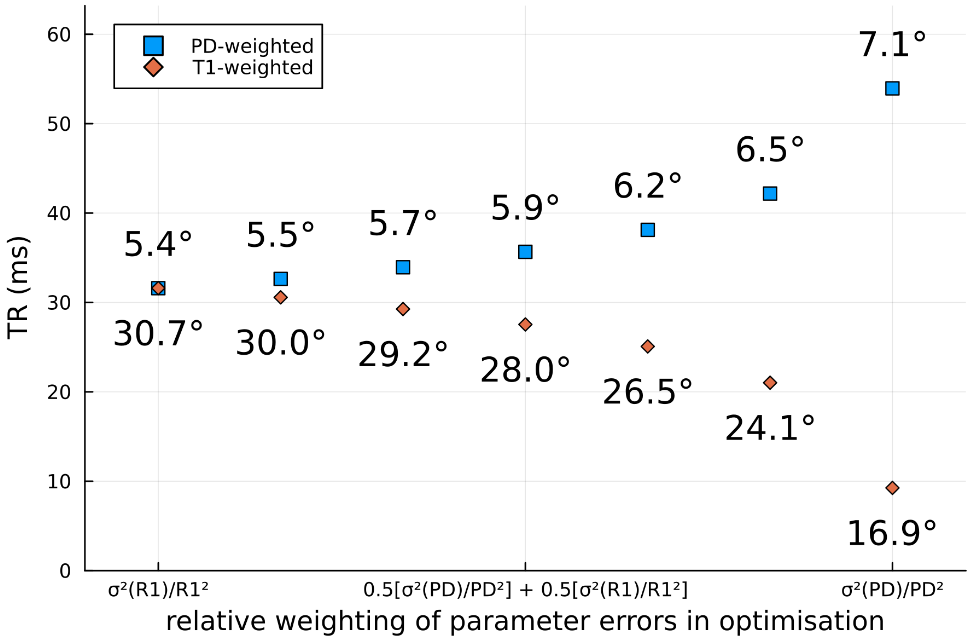

S2 Padé approximant in terms of sine and cosine

While the half-angle tangent form of the Padé approximant allows the R1 and A estimators to have the same form as in the small angle approximation case, we can also derive a $[1, 1]$ Padé approximant in R1 TR_n by starting from the more typical form of the Ernst equation expressed in terms of sine and cosine, Equation (1):

$$S_n \approx A \sin(\alpha_n) \frac{\mathrm{R1}\,\mathrm{TR}_n}{1 - \cos(\alpha_n) + \mathrm{R1}\,\mathrm{TR}_n[1 + \cos(\alpha_n)]/2}. \tag{S1}$$

Equation (S1) is equivalent to Equation (3), as can be shown by inserting the identities $\sin(\alpha_n) = \tau_n/(1 + \tau_n^2/4)$ and $\cos(\alpha_n) = (1 - \tau_n^2/4)/(1 + \tau_n^2/4)$.

Equation (S1) can then be solved for R1 and PD given data from two measurements S_1 and S_2 to give

$$\mathrm{R1} \approx 2 \frac{\{S_1[1 - \cos(\alpha_1)]\sin(\alpha_2)/\mathrm{TR}_1\} - \{S_2[1 - \cos(\alpha_2)]\sin(\alpha_1)/\mathrm{TR}_2\}}{S_2[1 + \cos(\alpha_2)]\sin(\alpha_1) - S_1[1 + \cos(\alpha_1)]\sin(\alpha_2)} \tag{S2}$$

and

$$A \approx \frac{1}{2} S_1 S_2 \frac{\mathrm{TR}_1[1 + \cos(\alpha_1)][1 - \cos(\alpha_2)] - \mathrm{TR}_2[1 - \cos(\alpha_1)][1 + \cos(\alpha_2)]}{S_2\mathrm{TR}_1 \sin(\alpha_1)[1 - \cos(\alpha_2)] - S_1\mathrm{TR}_2 \sin(\alpha_2)[1 - \cos(\alpha_1)]}. \tag{S3}$$

Inserting the above identities for $\sin(\alpha_n)$ and $\cos(\alpha_n)$ in terms of τ_n shows that these are equivalent to Equations (4) and (5). These estimators may have some small benefit in terms of floating point numerical accuracy as, unlike the tangent, sine and cosine are bounded, but such differences are far below the measurement accuracy in real experiments and so do not justify using these more complicated equations rather than Equations (4) and (5).

We note that Equation (S1) differs from the approximation found in Eq. (3) of Helms et al. which was derived as an intermediate step to the small angle approximation result by taking a Taylor expansion in R1 · TR of the numerator and denominator of Equation (1).[5] Figure S2 shows that while our [1/1] Padé approximant approach gives a good approximation of the signal, the approximation from Helms et al. shows a bias which propagates to the estimates of R1 and A.

Figure S2: Comparison of the approximation of the Ernst equation derived in this manuscript with an earlier approximation defined in Helms et al.[5] The simulations of the signal (top panel) used the same R1 and TR as the in vivo simulations in the main manuscript and the ground truth was computed using Equation (1). The approximations derived here maintain good accuracy over all angles, whereas the earlier approximation shows an angle-dependent bias. This angle dependent bias then propagates to estimates of R1 using the in vivo protocol (bottom panel). The novel approach used here does not show this bias, remaining accurate over all simulated angles and R1 values. Approx.: approximation.

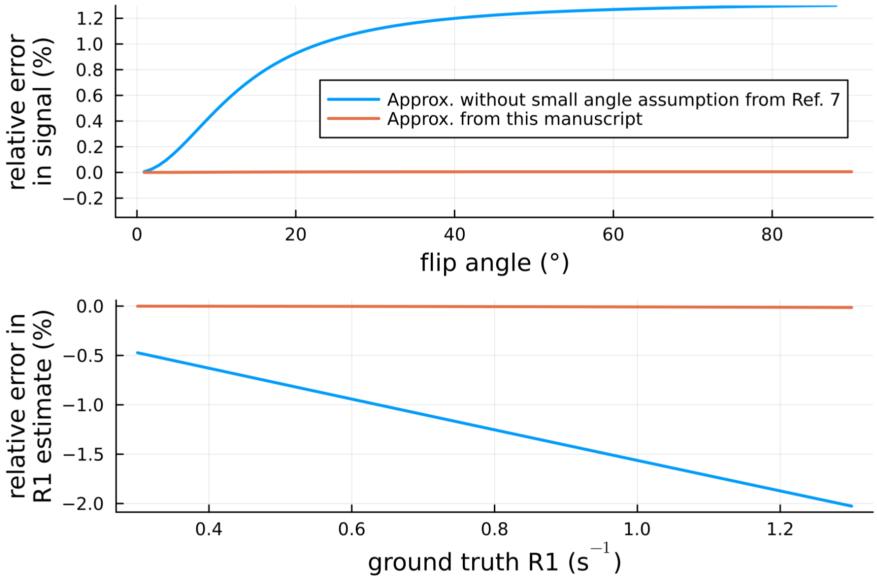

S3 Individual results for each in vivo participant and session

Below we show the data from all in vivo participants in Figures S3–S16. In each case the depicted slice from the second session was chosen to be the closest to that depicted for the first session, determined using the transform from applying `spm_coreg` from SPM12 to register the R2*-corrected PD-weighted volumes of the two sessions.

Figure S3: Application of the novel method (blue lines) improved estimation of R1 and A over the method assuming the small angle approximation (red lines) for participants 1–3 in line with theoretical predictions (Figure 1).

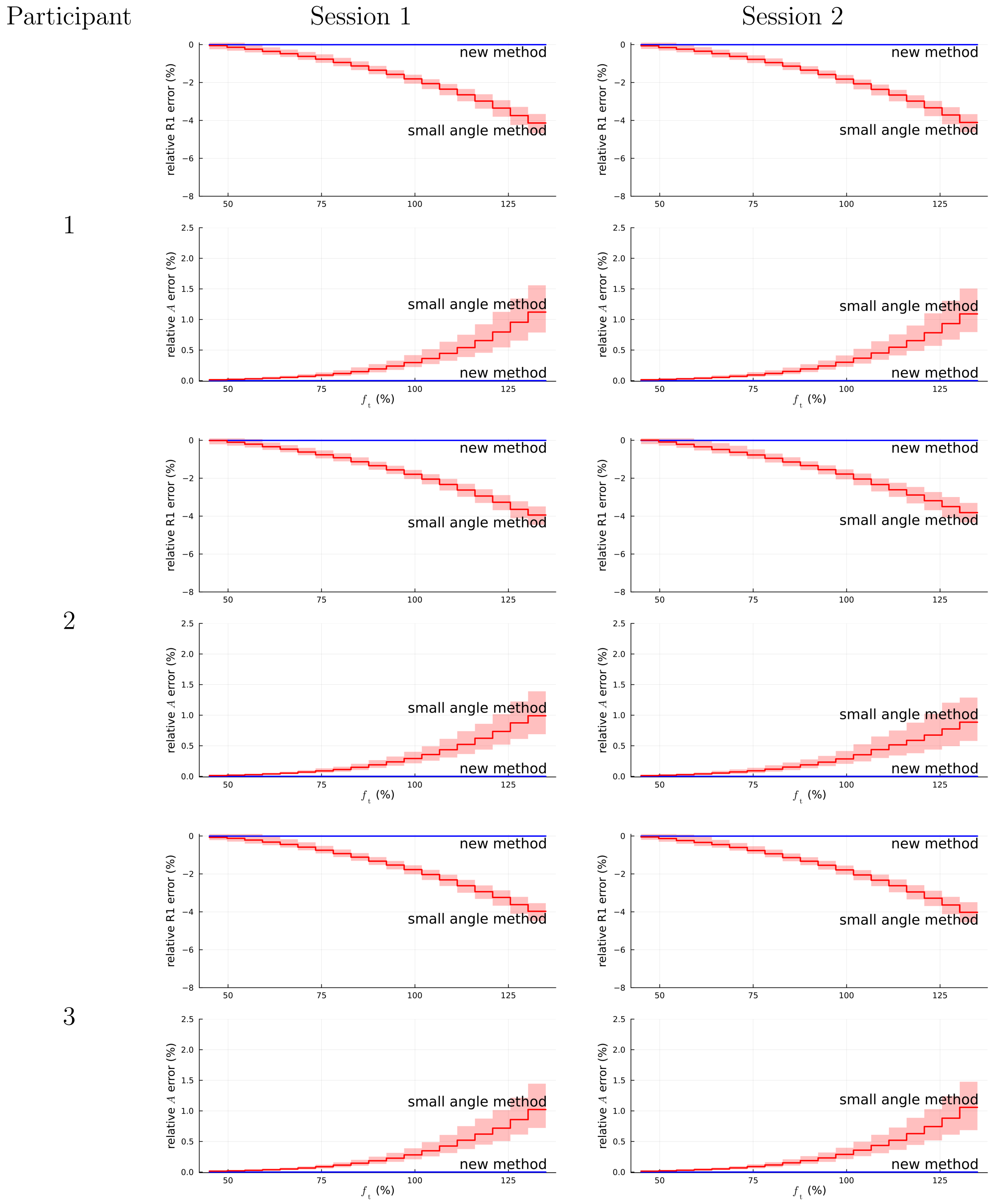

Figure S4: Application of the novel method (blue lines) improved estimation of R1 and A over the method assuming the small angle approximation (red lines) for participants 4–6 in line with theoretical predictions (Figure 1).

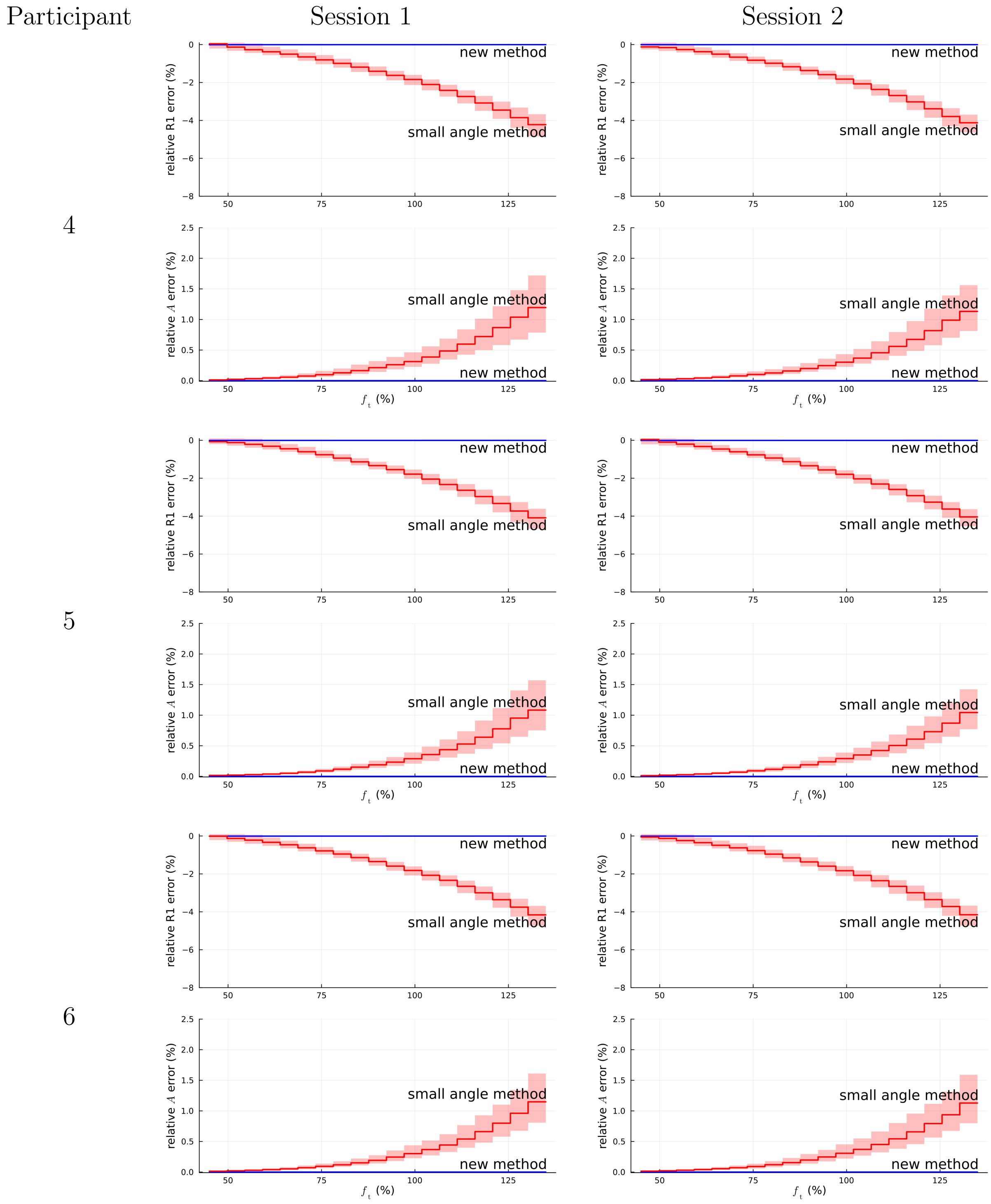

Figure S5: The spatial distribution of differences between small angle and novel estimator results followed the spatial distribution of the f_t map in participant 1, session 1. A: R1 and B: A maps estimated using the novel estimators in an exemplary slice. C and D: Relative differences of small angle and novel estimator results show the same spatial pattern as E: the f_t map (interpolated to MPM space). F: Histograms of the f_t-dependence of the errors over WM (brighter colour means more voxels in a bin). abs.: absolute value; a.u.: arbitrary units; L: left; P: posterior.

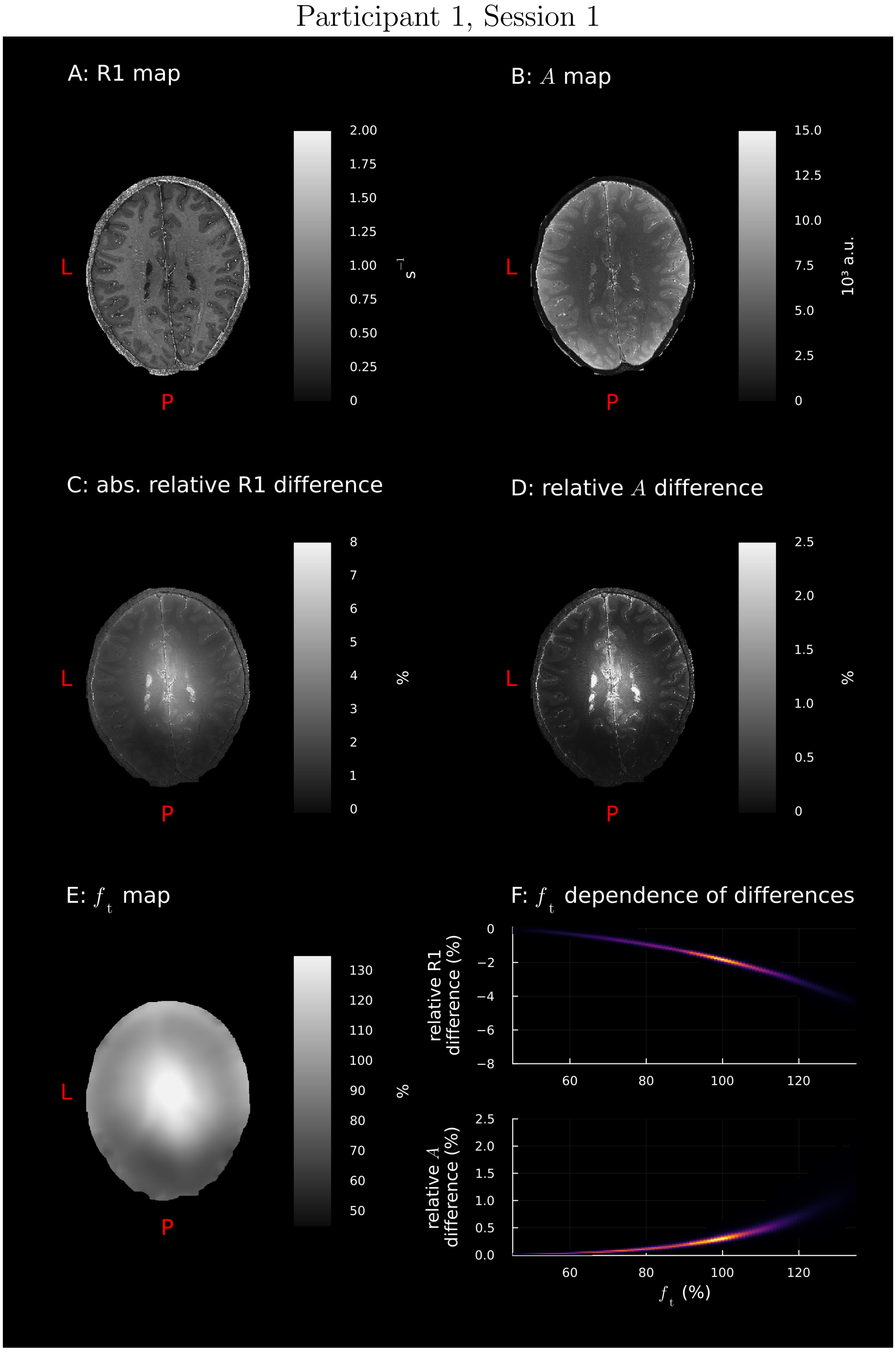

Figure S6: The spatial distribution of differences between small angle and novel estimator results followed the spatial distribution of the f_t map in participant 1, session 2. A: R1 and B: A maps estimated using the novel estimators in an exemplary slice. C and D: Relative differences of small angle and novel estimator results show the same spatial pattern as E: the f_t map (interpolated to MPM space). F: Histograms of the f_t-dependence of the errors over WM (brighter colour means more voxels in a bin). abs.: absolute value; a.u.: arbitrary units; L: left; P: posterior.

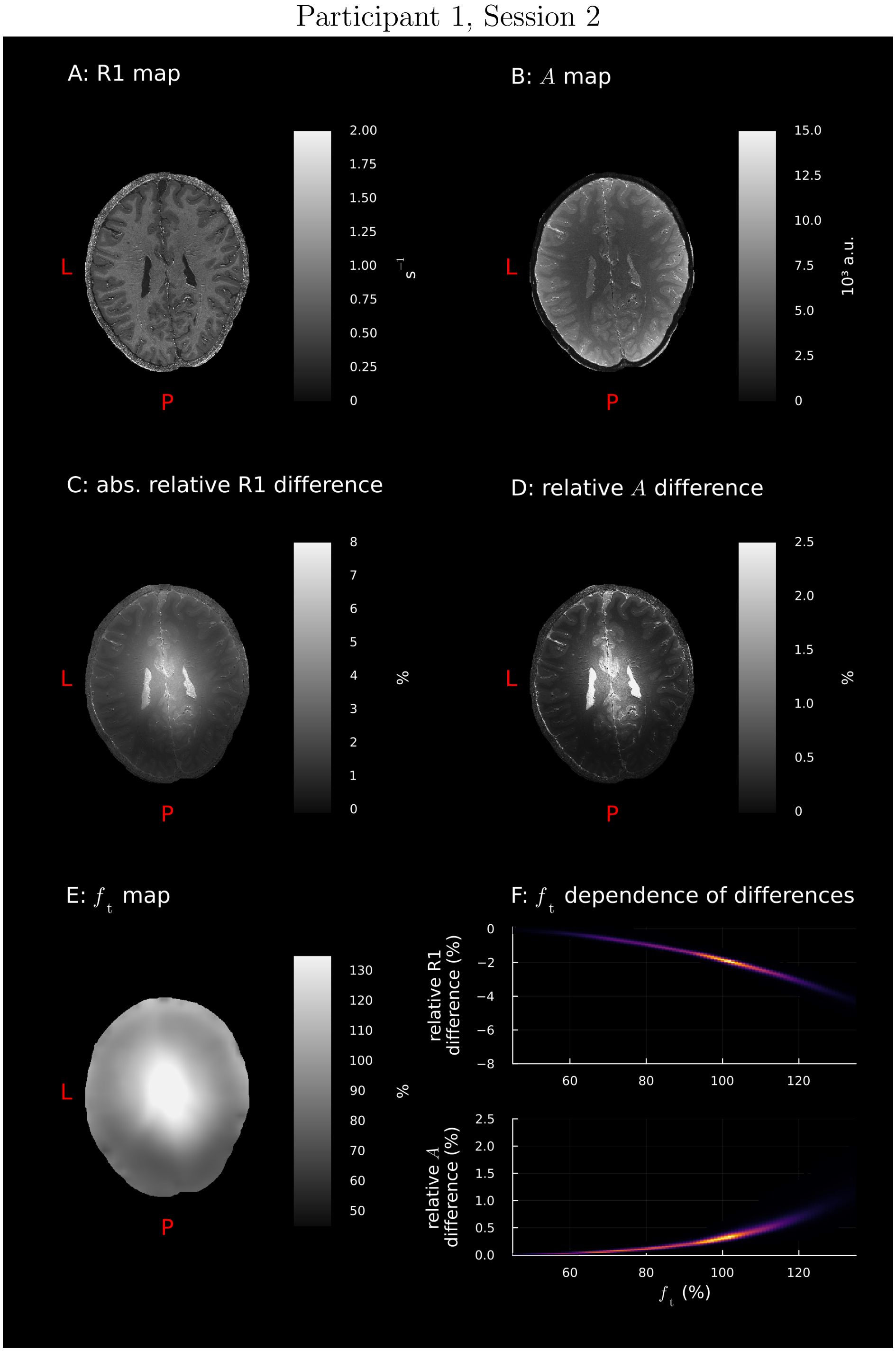

Figure S7: The spatial distribution of differences between small angle and novel estimator results followed the spatial distribution of the f_t map in participant 2, session 1. A: R1 and B: A maps estimated using the novel estimators in an exemplary slice. C and D: Relative differences of small angle and novel estimator results show the same spatial pattern as E: the f_t map (interpolated to MPM space). F: Histograms of the f_t-dependence of the errors over WM (brighter colour means more voxels in a bin). abs.: absolute value; a.u.: arbitrary units; L: left; P: posterior.

Participant 2, Session 1

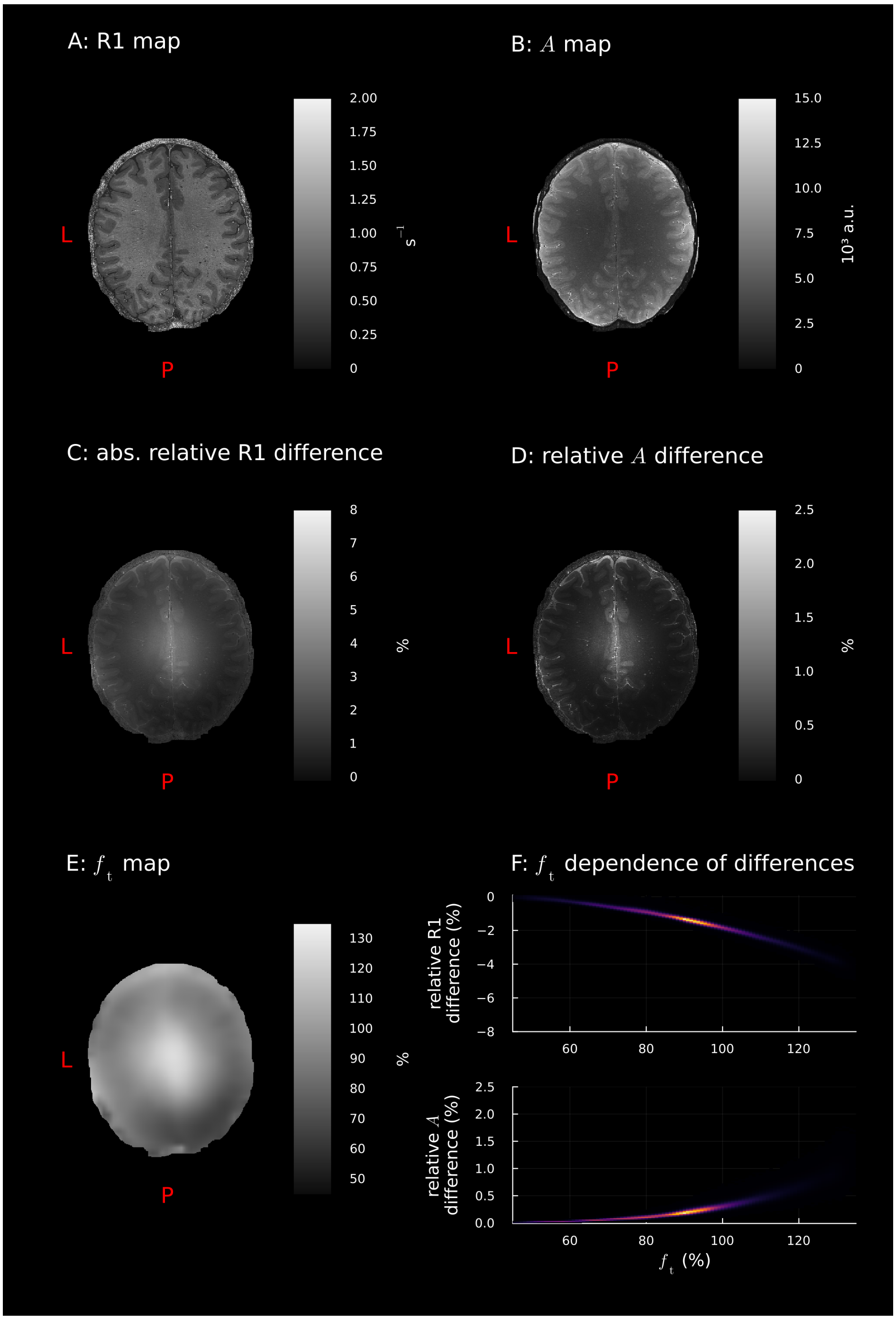

Figure S8: The spatial distribution of differences between small angle and novel estimator results followed the spatial distribution of the f_t map in participant 2, session 2. A: R1 and B: A maps estimated using the novel estimators in an exemplary slice. A ripple artefact can be seen in the R1 and A maps which is probably related to dynamic B0 inhomogeneity. C and D: Relative differences of small angle and novel estimator results show the same spatial pattern as E: the f_t map (interpolated to MPM space). F: Histograms of the f_t-dependence of the errors over WM (brighter colour means more voxels in a bin). abs.: absolute value; a.u.: arbitrary units; L: left; P: posterior.

Participant 2, Session 2

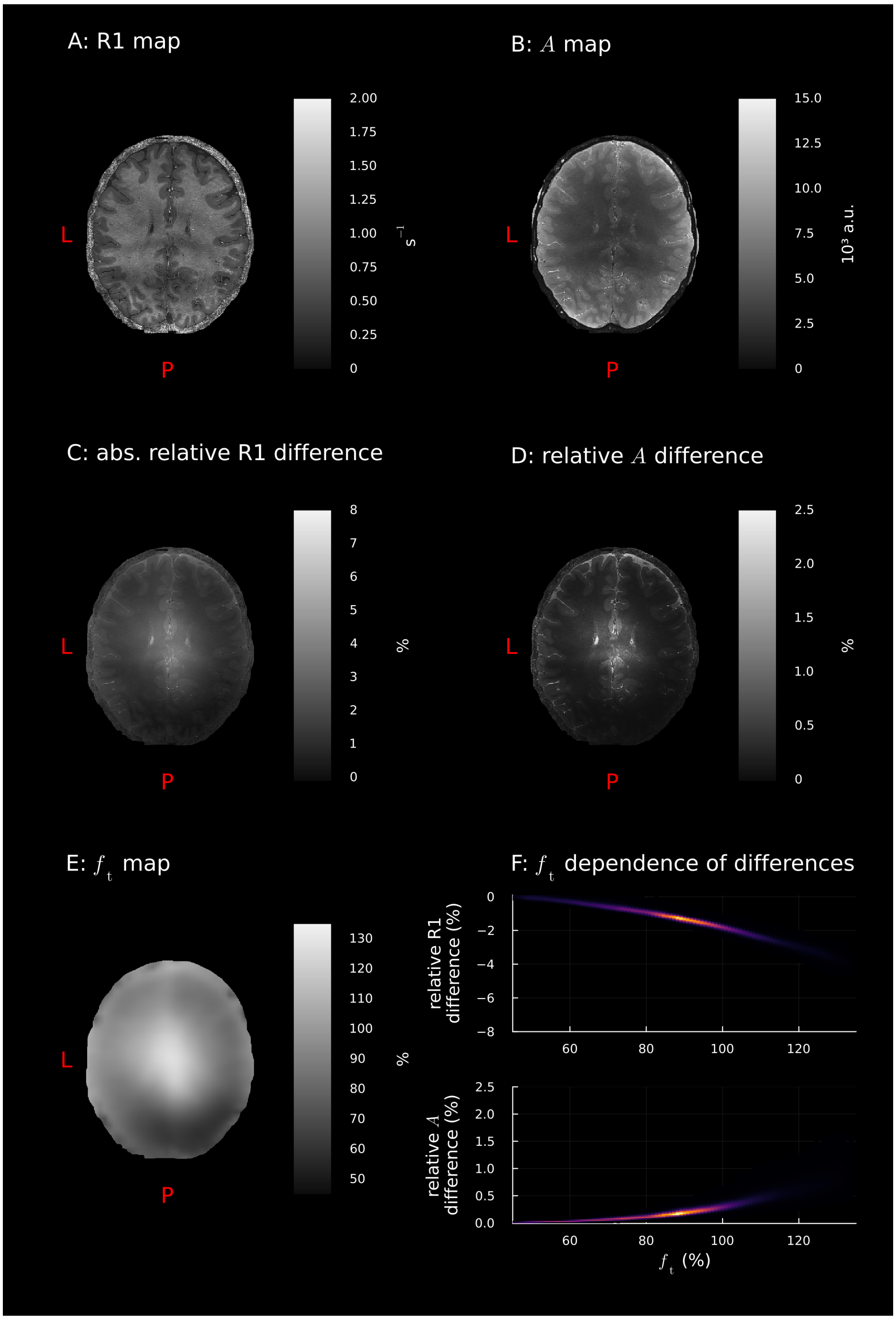

Figure S9: The spatial distribution of differences between small angle and novel estimator results followed the spatial distribution of the f_t map in participant 3, session 1. A: R1 and B: A maps estimated using the novel estimators in an exemplary slice. C and D: Relative differences of small angle and novel estimator results show the same spatial pattern as E: the f_t map (interpolated to MPM space). F: Histograms of the f_t-dependence of the errors over WM (brighter colour means more voxels in a bin). abs.: absolute value; a.u.: arbitrary units; L: left; P: posterior.

Participant 3, Session 1

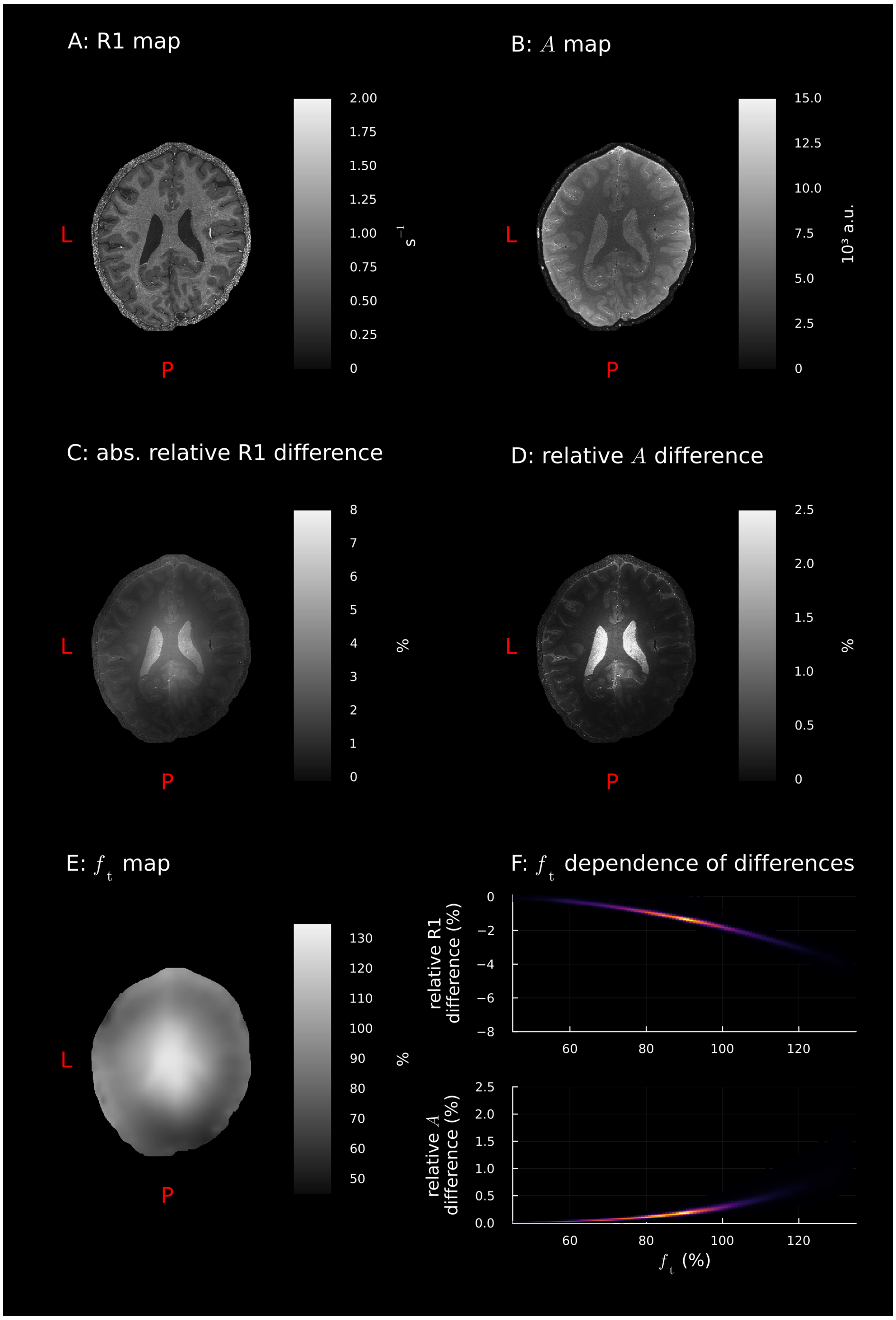

Figure S10: The spatial distribution of differences between small angle and novel estimator results followed the spatial distribution of the f_t map in participant 3, session 2. A: R1 and B: A maps estimated using the novel estimators in an exemplary slice. C and D: Relative differences of small angle and novel estimator results show the same spatial pattern as E: the f_t map (interpolated to MPM space). F: Histograms of the f_t-dependence of the errors over WM (brighter colour means more voxels in a bin). abs.: absolute value; a.u.: arbitrary units; L: left; P: posterior.

Participant 3, Session 2

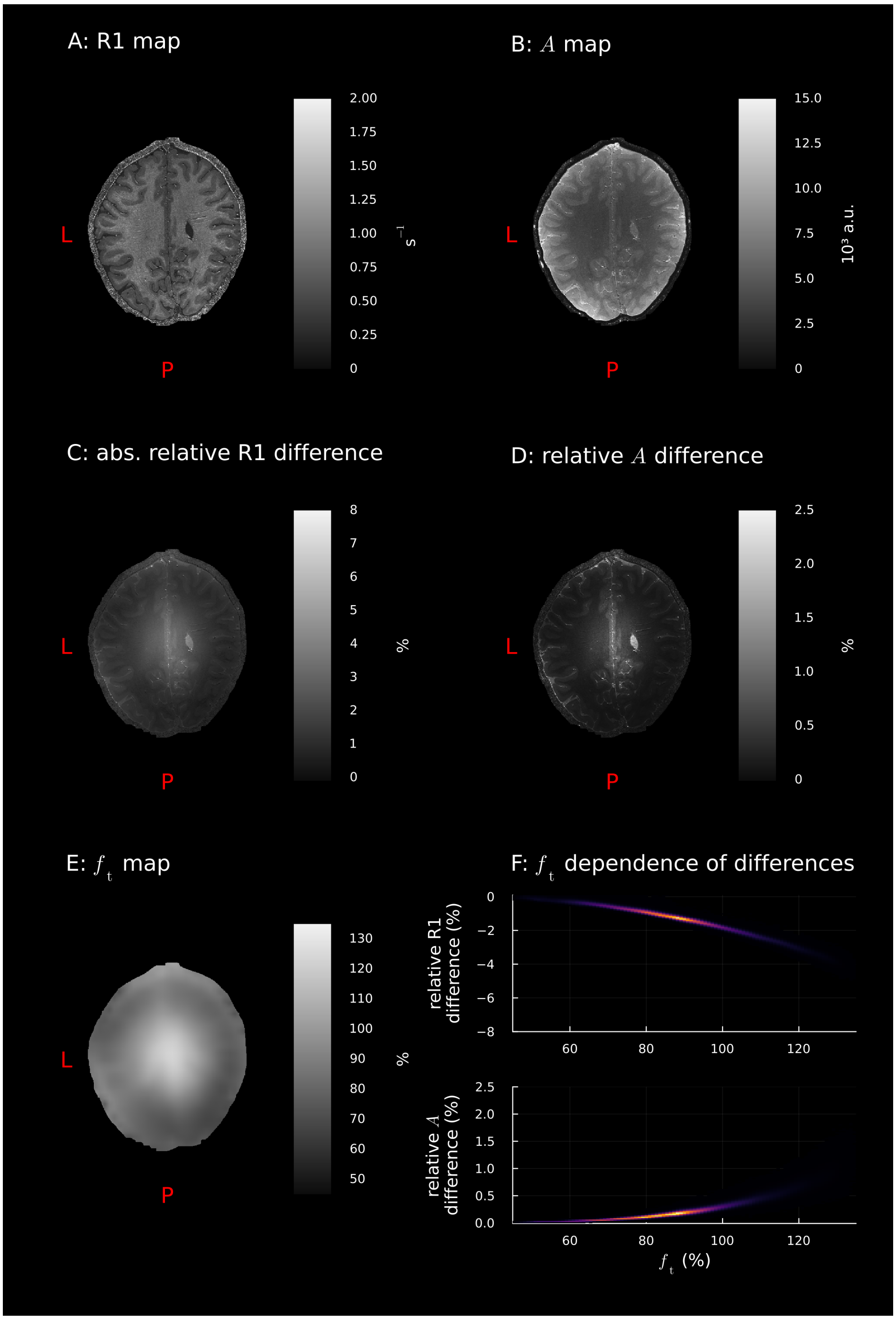

Figure S11: The spatial distribution of differences between small angle and novel estimator results followed the spatial distribution of the f_t map in participant 4, session 1. A: R1 and B: A maps estimated using the novel estimators in an exemplary slice. C and D: Relative differences of small angle and novel estimator results show the same spatial pattern as E: the f_t map (interpolated to MPM space). F: Histograms of the f_t-dependence of the errors over WM (brighter colour means more voxels in a bin). abs.: absolute value; a.u.: arbitrary units; L: left; P: posterior.

Participant 4, Session 1

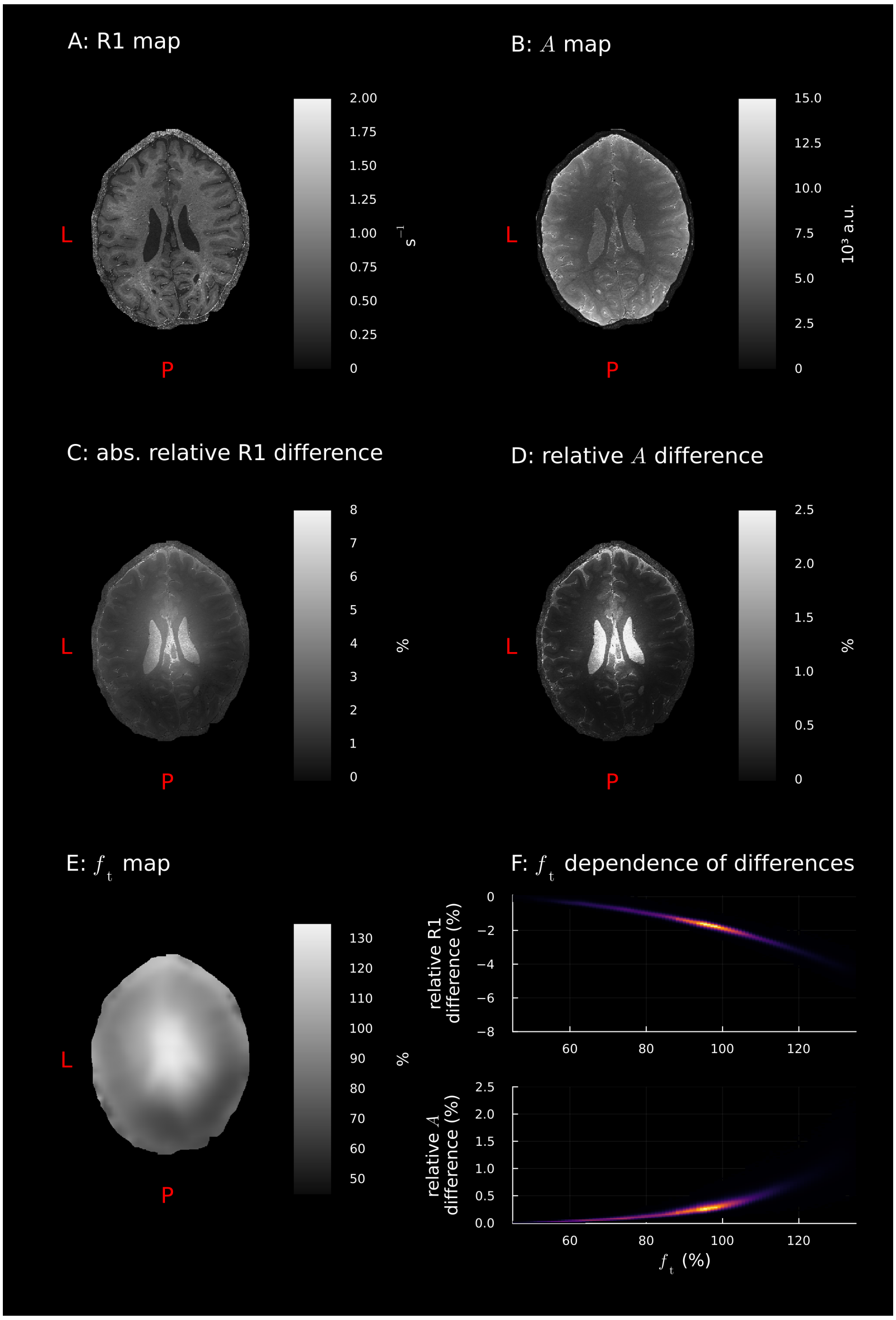

Figure S12: The spatial distribution of differences between small angle and novel estimator results followed the spatial distribution of the f_t map in participant 4, session 2. A: R1 and B: A maps estimated using the novel estimators in an exemplary slice. C and D: Relative differences of small angle and novel estimator results show the same spatial pattern as E: the f_t map (interpolated to MPM space). F: Histograms of the f_t-dependence of the errors over WM (brighter colour means more voxels in a bin). abs.: absolute value; a.u.: arbitrary units; L: left; P: posterior.

Participant 4, Session 2

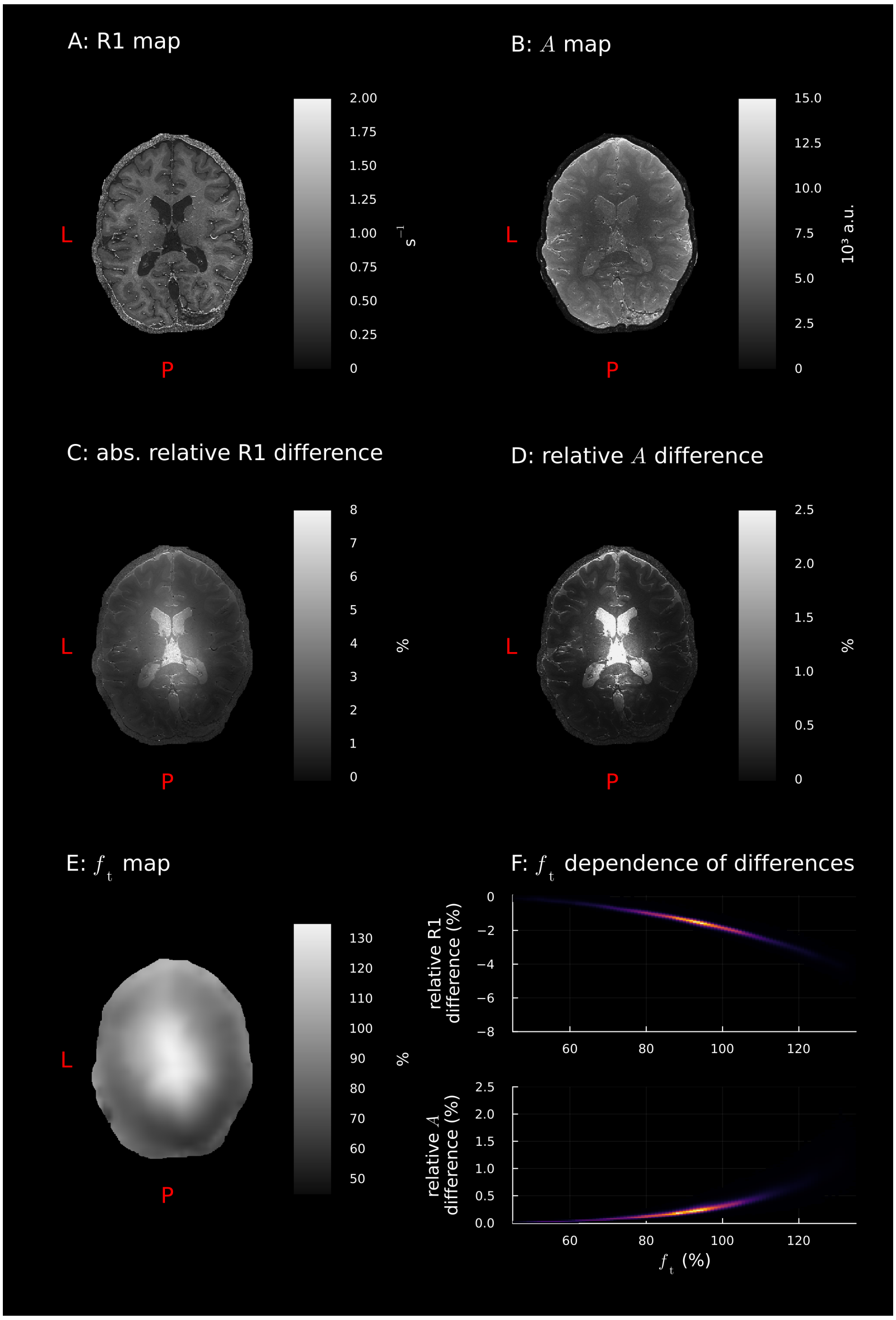

Figure S13: The spatial distribution of differences between small angle and novel estimator results followed the spatial distribution of the f_t map in participant 5, session 1. A: R1 and B: A maps estimated using the novel estimators in an exemplary slice. C and D: Relative differences of small angle and novel estimator results show the same spatial pattern as E: the f_t map (interpolated to MPM space). F: Histograms of the f_t-dependence of the errors over WM (brighter colour means more voxels in a bin). abs.: absolute value; a.u.: arbitrary units; L: left; P: posterior.

Participant 5, Session 1

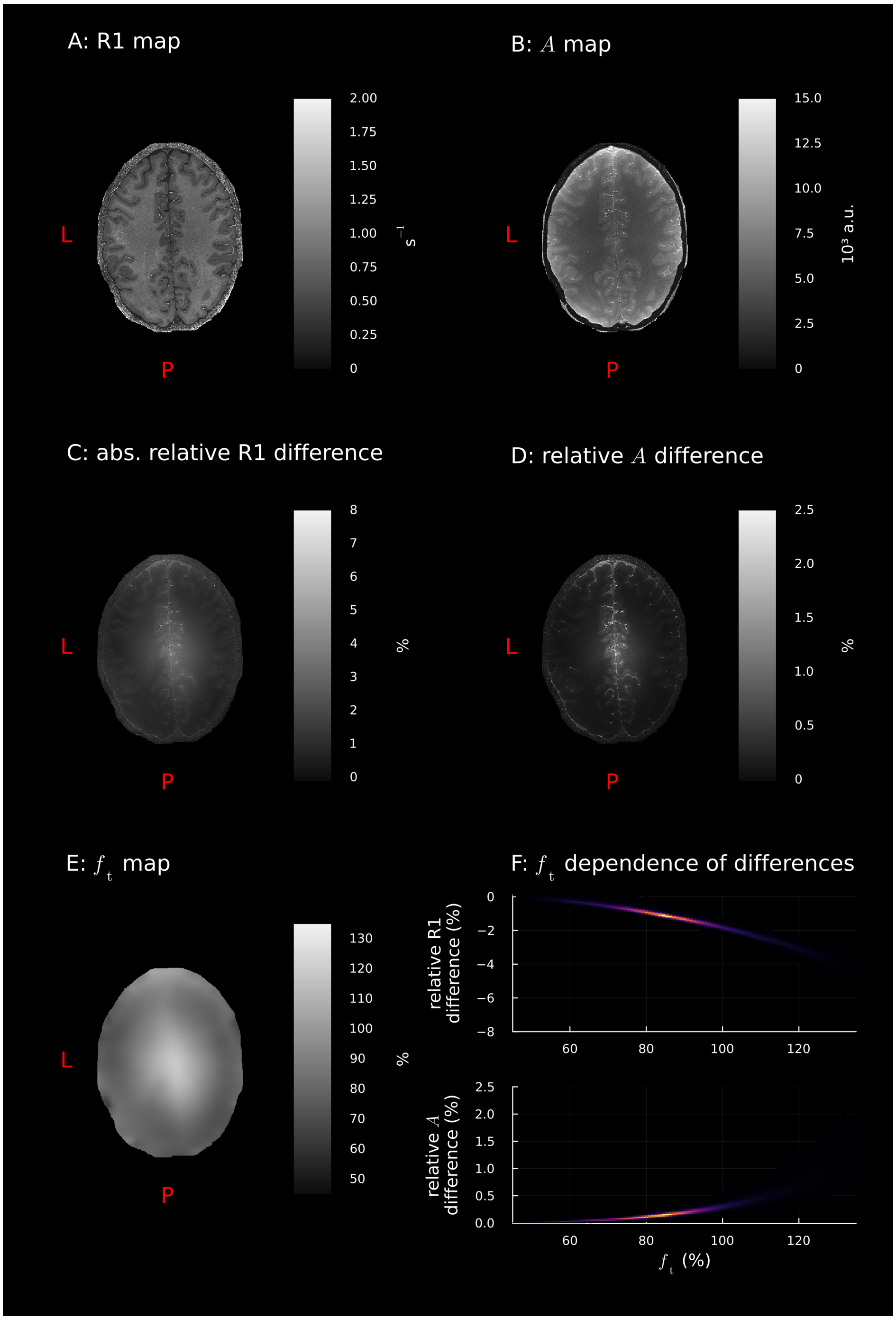

Figure S14: The spatial distribution of differences between small angle and novel estimator results followed the spatial distribution of the f_t map in participant 5, session 2. A: R1 and B: A maps estimated using the novel estimators in an exemplary slice. C and D: Relative differences of small angle and novel estimator results show the same spatial pattern as E: the f_t map (interpolated to MPM space). F: Histograms of the f_t-dependence of the errors over WM (brighter colour means more voxels in a bin). abs.: absolute value; a.u.: arbitrary units; L: left; P: posterior.

Participant 5, Session 2

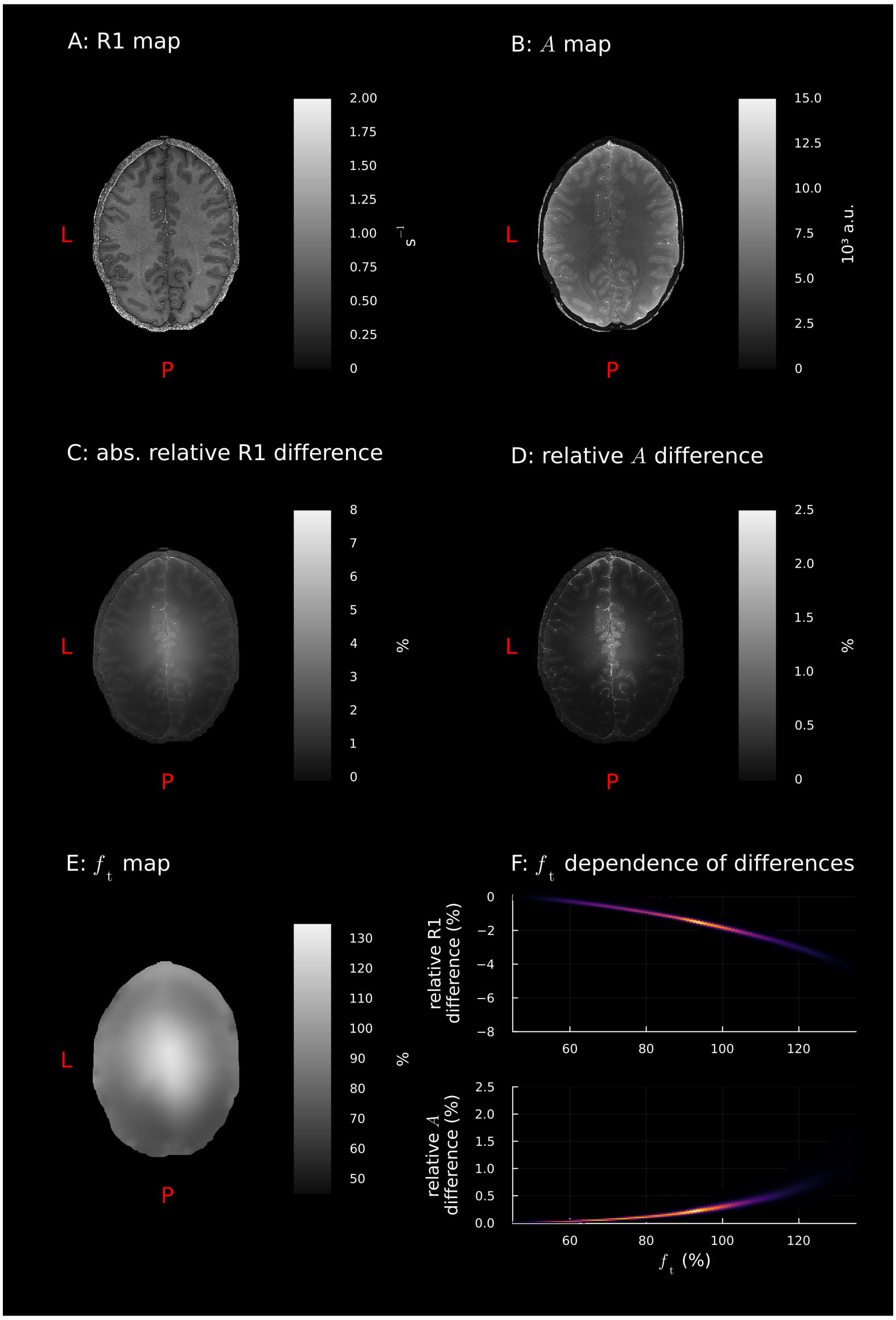

Figure S15: The spatial distribution of differences between small angle and novel estimator results followed the spatial distribution of the f_t map in participant 6, session 1. A: R1 and B: A maps estimated using the novel estimators in an exemplary slice. C and D: Relative differences of small angle and novel estimator results show the same spatial pattern as E: the f_t map (interpolated to MPM space). F: Histograms of the f_t-dependence of the errors over WM (brighter colour means more voxels in a bin). abs.: absolute value; a.u.: arbitrary units; L: left; P: posterior.

Participant 6, Session 1

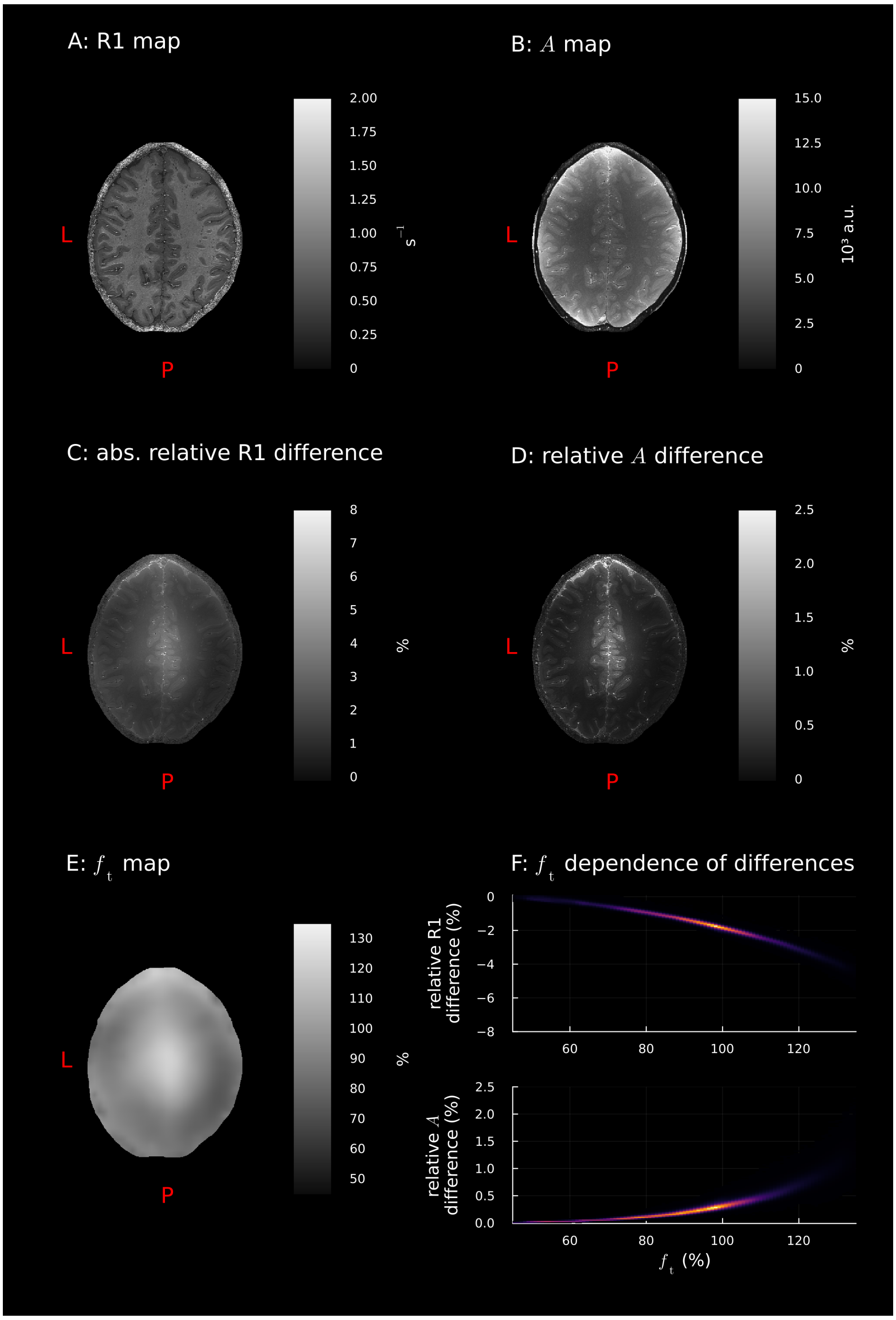

Figure S16: The spatial distribution of differences between small angle and novel estimator results followed the spatial distribution of the f_t map in participant 6, session 2. A: R1 and B: A maps estimated using the novel estimators in an exemplary slice. C and D: Relative differences of small angle and novel estimator results show the same spatial pattern as E: the f_t map (interpolated to MPM space). F: Histograms of the f_t-dependence of the errors over WM (brighter colour means more voxels in a bin). abs.: absolute value; a.u.: arbitrary units; L: left; P: posterior.

Participant 6, Session 2

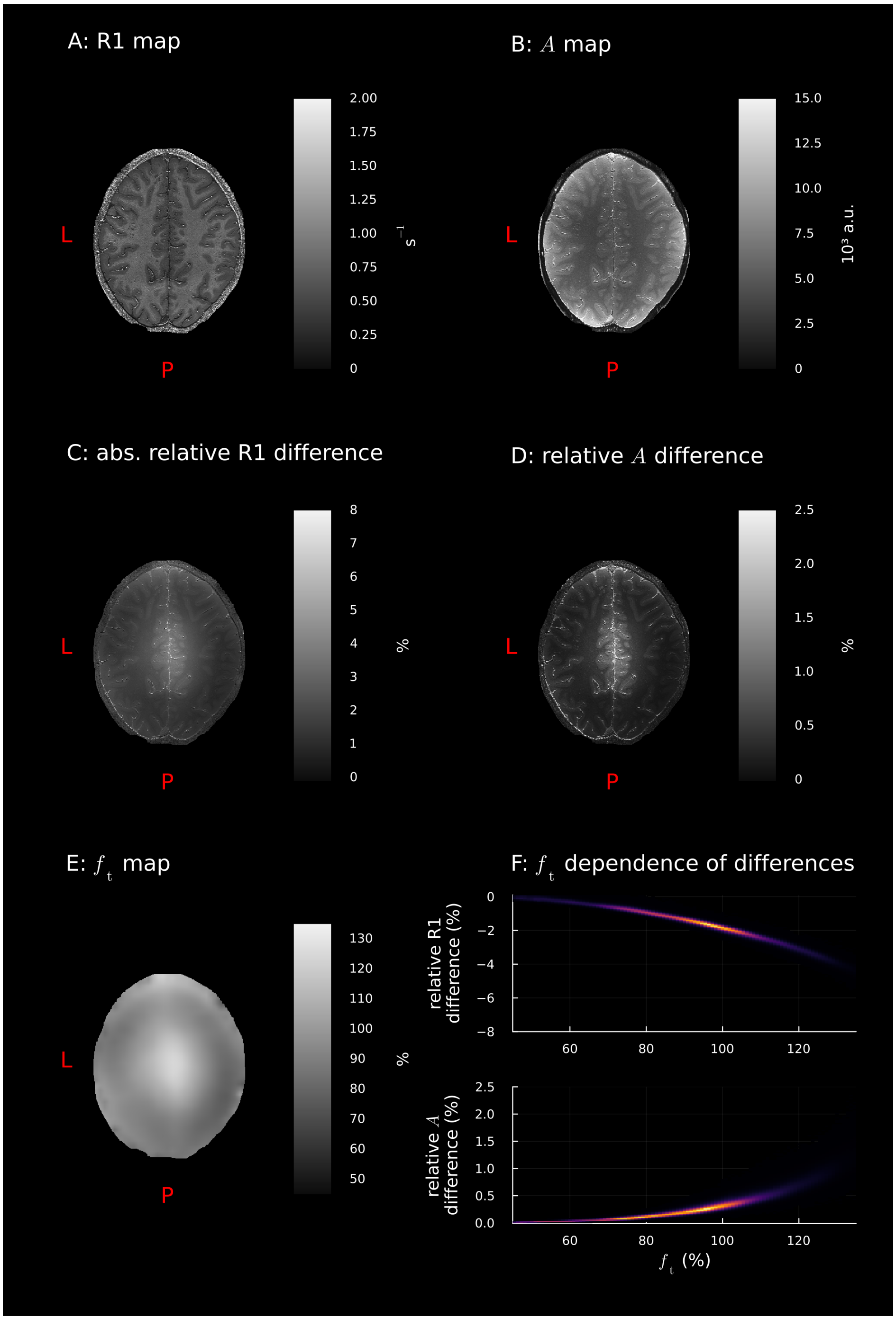

S4 Imperfect spoiling does not appreciably change the results for the in vivo and postmortem protocols

We used an implementation of the R1 correction method from Preibisch et al.[6] extended to include diffusion spoiling[7] to investigate the impact of imperfect spoiling correction on our results. In brief, the correction method simulates the spin dynamics of spins with given R1, T2, diffusivity and f_t values (in this implementation using the EPG-X toolbox[8]) and then estimates a polynomial in f_t and estimated R1 which can be used to correct the estimated R1 for imperfect spoiling. Instead of R1 being estimated using an equation assuming the small angle approximation[6] or an exact estimator assuming equal TRs, we adapted the method to use Equation (4) so that it is consistent with our estimated R1 values. This method has been implemented in the open source hMRI toolbox and is available from v0.2.5 and later (`hmri.info`).

S4.1 In vivo

For our simulations of the in vivo protocol we chose 16 R1 values to cover the range of R1 in WM and GM at 7T from Rooney et al.,[3] 17 f_t values from 50 % to 130 %, and the diffusion constant and T2 were taken from the parameters used for simulation 2 in Corbin et al.[7]

The root mean square error between the ground truth R1 and R1 estimated using the novel method calculated from the simulations to generate imperfect spoiling coefficients was 0.456 %, implying that the effect of imperfect spoiling is very small for this protocol – much smaller than the bias due to use of the small angle approximation. This is in line with Figures S17 and S18, which show that imperfect spoiling correction does not affect our results that the novel method improved estimation of R1. The results for the other participants were similar and so are not reproduced here.

Figure S17: The spatial distribution of differences in R1 between small angle and novel estimator results still followed the spatial distribution of the f_t map in participant 1, session 1 after correcting for imperfect spoiling. A: R1 map estimated using the novel estimators in an exemplary slice. B: Relative differences of small angle and novel estimator results show the same spatial pattern as C: the f_t map (interpolated to MPM space). D: Histogram of the f_t-dependence of the errors over WM (brighter colour means more voxels in a bin). abs.: absolute value; a.u.: arbitrary units; L: left; P: posterior.

Participant 1, Session 1

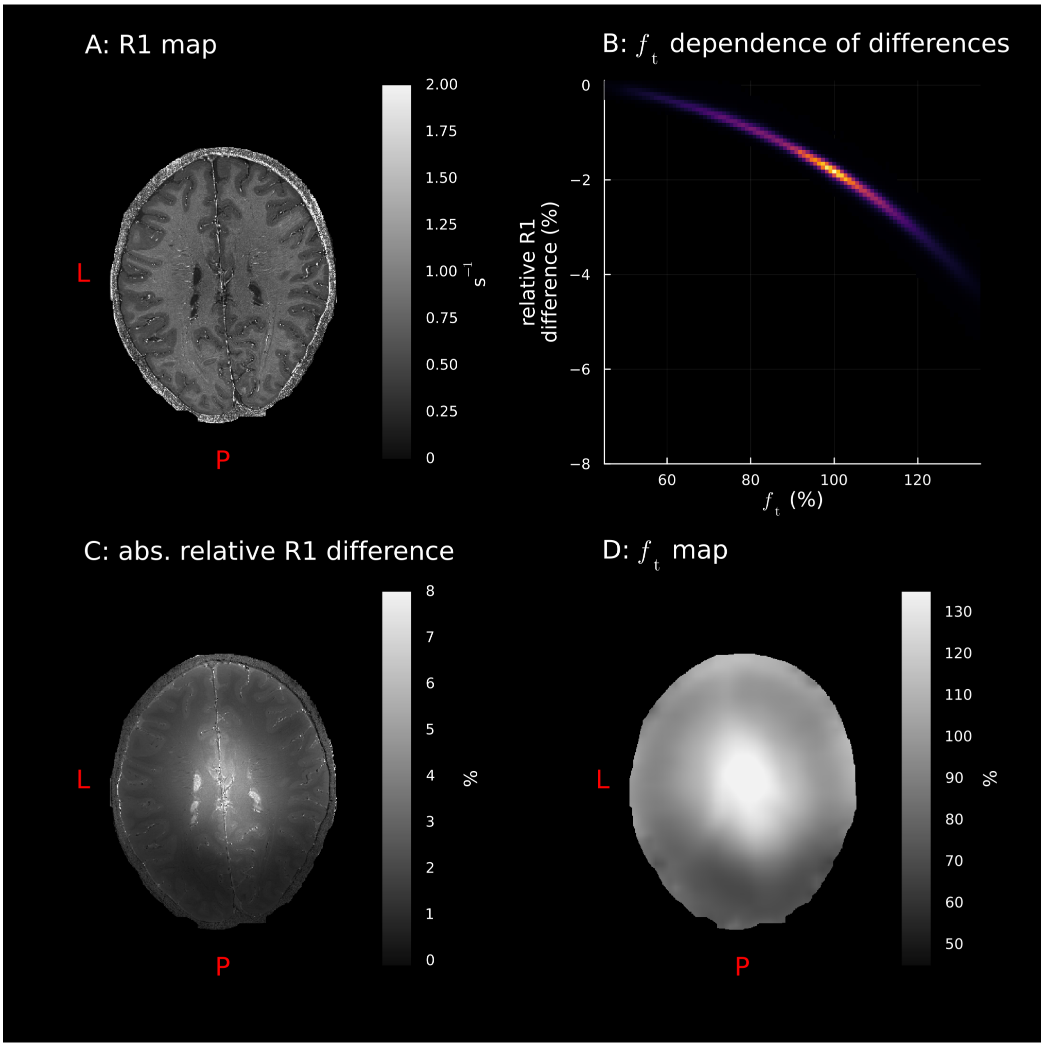

Figure S18: The spatial distribution of differences in R1 between small angle and novel estimator results still followed the spatial distribution of the f_t map in participant 1, session 2 after correcting for imperfect spoiling. A: R1 map estimated using the novel estimators in an exemplary slice. B: Relative differences of small angle and novel estimator results show the same spatial pattern as C: the f_t map (interpolated to MPM space). D: Histogram of the f_t-dependence of the errors over WM (brighter colour means more voxels in a bin). abs.: absolute value; a.u.: arbitrary units; L: left; P: posterior.

Participant 1, Session 2

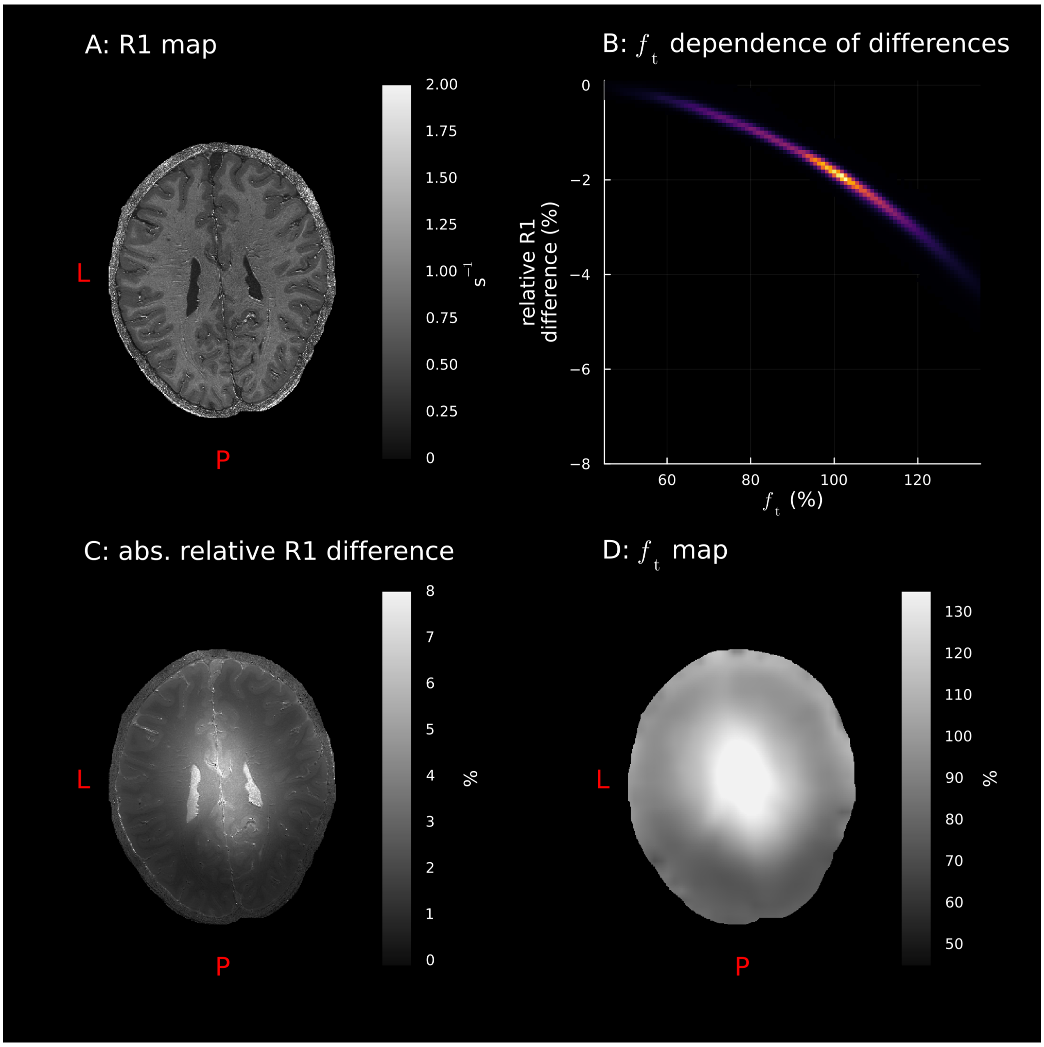

S4.2 Postmortem

For our simulations of the postmortem protocol the diffusion constant was estimated as mean diffusivity $\sim 0.2\,\mu\text{m}^2/\text{ms}$ from the data provided by Eichner et al.,[9,10] $T2 = 30\,\text{ms}$ was chosen as a compromise between grey matter values ($\sim 40\,\text{ms}$) and white matter values ($\sim 20\,\text{ms}$) estimated from a postmortem chimpanzee brain fixed using the same procedure as the brain used in the main manuscript (data not shown; values kindly provided by Felix Büttner), and we chose 16 R1 values between $0.5\,\text{s}^{-1}$ to $2\,\text{s}^{-1}$, covering the range observed in the data, and 17 f_t values from 50 % to 130 %.

The root mean square error between the ground truth R1 and R1 estimated using the novel method calculated from the simulations to generate imperfect spoiling coefficients was 0.52 %, implying that the effect of imperfect spoiling is also very small for the postmortem protocol – again much smaller than the bias due to use of the small angle approximation. This is in line with Figure S19, which shows that imperfect spoiling correction does not strongly affect the result that the novel method improved estimation of R1 in this case.

Figure S19: The spatial distribution of differences in R1 between small angle and novel estimator results still followed the spatial distribution of the f_t map in the postmortem chimpanzee brain after correcting for imperfect spoiling. A: R1 map estimated using the novel estimators in an exemplary slice. B: Relative differences of the small angle estimates to the novel estimator results show the same spatial pattern as C: the f_t map (interpolated to MPM space). D: Histogram of the f_t-dependence of the errors over the brain (brighter colour means more voxels in a bin). The brain is slightly rotated in plane as it was not perfectly aligned to the acquired field of view. abs.: absolute value; a.u.: arbitrary units; L: left; P: posterior.

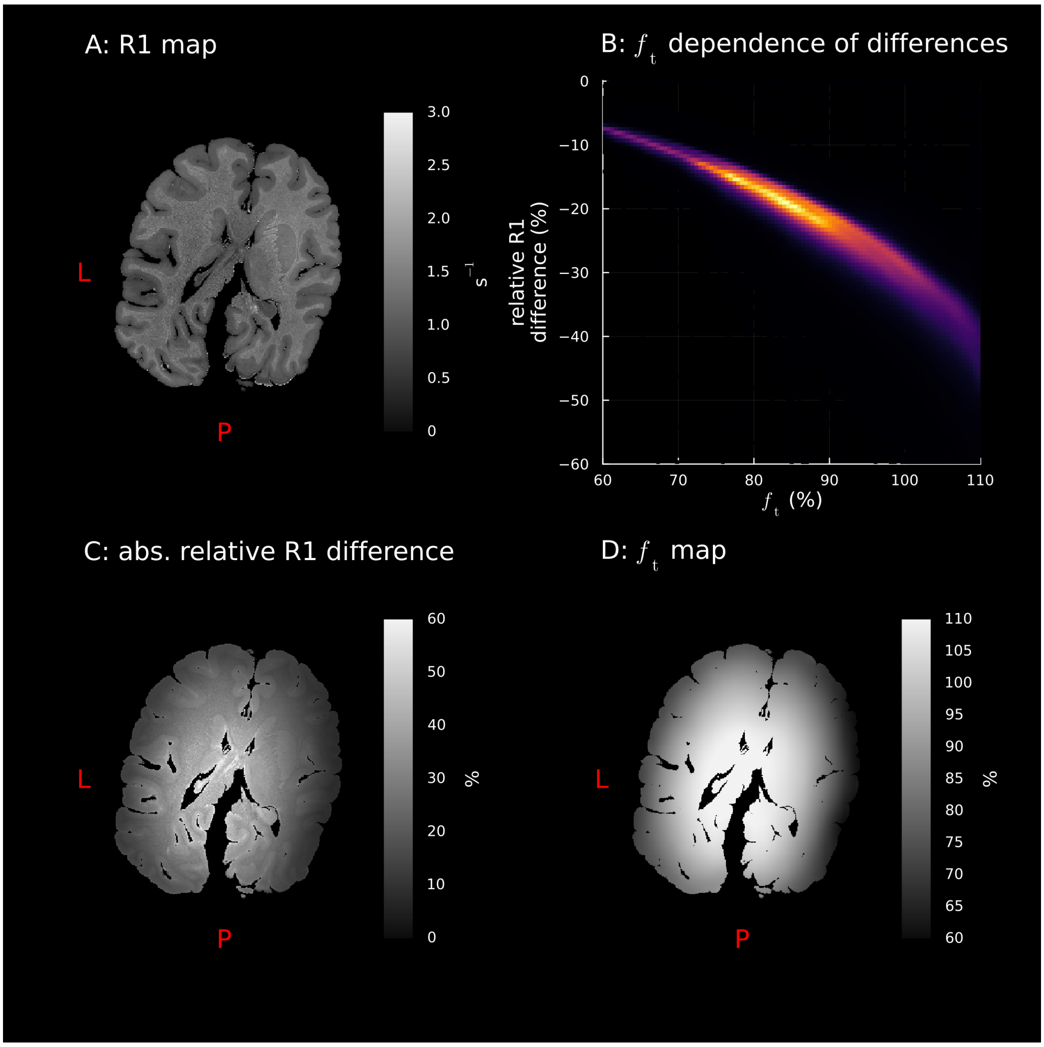

S5 The variance of the quantitative maps is similar for both the novel method and the method assuming the small angle approximation

Figure S20: Simulations showing mean (line) and first order propagation of error estimates of standard deviation (shaded area) of R1 and A for the in vivo and postmortem protocols at typical SNR values estimated from the data. In all cases the variance of the new method is no worse than the small angle method, however the bias in the small angle approximation causes extreme outliers to become more likely. Rough estimates of typical SNR in vivo (18) and postmortem (51) were estimated at $f_t \approx 100\,\%$ from the respective PD-weighted data using the R2* fit residuals following Mohammadi et al.[2] These simulations likely overestimate the variance at lower f_t as they do not include the effects of receive B1, which in practice will tend to counteract the decrease in SNR as f_t decreases due to the approximate inverse relationship of transmit and receive B1 inhomogeneity.

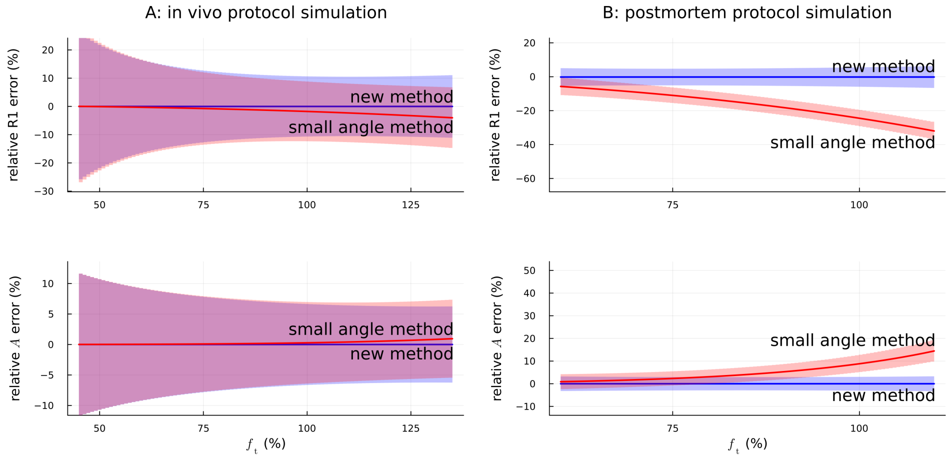

S6 Phantom tests show that the novel method is also accurate when TRs are unequal

A phantom containing 40 % polyvinylpyrrolidone was scanned on a Siemens Prisma^{fit} 3T scanner (Siemens Healthcare, Erlangen, Germany) with two different protocols reflecting optimal parameters for R1 and A precision, respectively. For the selection of optimal parameters we assumed R1 = 1 s^{-1} based on literature values[11] and fixed $\text{TR}_1 + \text{TR}_2 =$ 60 ms, allowing time for multiple echoes in at least one of TR_1 and TR_2. The optimal parameters for estimating R1 were ${\alpha_1}^{(\text{nominal})} = 6°$, ${\alpha_2}^{(\text{nominal})} = 33°$ and $\text{TR}_1 = \text{TR}_2 = 30$ ms. For A they were ${\alpha_1}^{(\text{nominal})} = 8°$, ${\alpha_2}^{(\text{nominal})} = 18°$, $\text{TR}_1 = 51.2$ ms and $\text{TR}_2 = 8.8$ ms. Each protocol consisted of two gradient and RF spoiled multi-echo 3D gradient echo FLASH datasets (1 mm isotropic resolution, number of sampled echo times limited by TR such that TR = 8.8 ms: one TE 2.3 ms, TR $\geq$ 30 ms: 10 equispaced TE from 2.3 ms to 23.0 ms, bandwidth: 488 Hz/pixel, matrix size: 224/256/176 (phase1/read/phase2), CAIPIRINHA partially parallel imaging factor[12] 2 in each phase-encoding direction, integrated k-space reference lines: 40/40 (phase1/phase2), 6π per pixel gradient spoiling at 33.3 mT m^{-1} gradient strength per TR, RF spoiling increment 137°, acquisition time: 6 minutes for each dataset).

To test the impact of different f_t mapping methods, data were acquired using both 3D-EPI SE/STE[13] (4 mm isotropic resolution, 11 equispaced spin echo flip angles from 130° to 330°, mixing time: 33.8 ms) and 3D-actual flip angle imaging[14] (AFI; 4 mm isotropic resolution, TE: 2.84 ms, flip angle: 60°, bandwidth: 425 Hz/pixel, matrix size: 48/56/64 (phase1/read/phase2), partially parallel imaging factor[15] 3 in the first phase encoding direction, 18 integrated k-space reference lines, $\text{TR}_2/\text{TR}_1 = 150\,\text{ms}/50\,\text{ms}$ with respective spoiler moments 78 mT ms m^{-1}/26 mT ms m^{-1} at spoiler gradient amplitude 26 mT m^{-1}, additional diffusion spoiling in each TR lasting $\delta = 42$ ms split into periods $(\delta/4, \delta/2, \delta/4)$ with gradient polarity $(+, -, +)$, RF spoiling increments were varied between TRs:[16] the increment for TR_2 was ϕ and for TR_1 was $\text{TR}_1\phi/\text{TR}_2$, with $\phi = 50°$ chosen based on simulations of the protocol using the EPG-X toolbox[8]).

An inversion recovery R1 mapping protocol was also recorded to provide ground truth R1 values in a single slice (4 mm isotropic resolution, TR 6000 ms, TE 16 ms, 10 inversion times: 30 ms, 50 ms, 100 ms, 170 ms, 310 ms, 560 ms, 1000 ms, 1790 ms, 3190 ms and 5900 ms, matrix size: 64/48/1 (read/phase/slice)).

Maps of R1 and A were calculated using the hMRI toolbox,[17] using only the first echo of each dataset to allow comparability between the different protocols. The calculation was performed for each protocol and f_t mapping method using the novel estimation method and the method assuming the small angle approximation. f_t map smoothing was disabled to preserve the resolution of the f_t maps, registration between the datasets was disabled as the phantom did not move, and A receive bias correction and calibration[17]

was disabled to avoid it confounding the analysis. The inversion recovery data was fitted using qMRLab (v2.4.1).[18,19]

It can be seen from Figures S21–S24 that the bias from using the small angle approximation is removed in all cases by using the novel method. The magnitude of the bias is smaller in the A-optimal case (Figures S23 and S24) because the maximum flip angle is smaller. Figures S25 and S26 show the good agreement between R1 measured using the two protocols.

There was good agreement between the AFI and SE/STE median R1 estimates of $1.18\,\mathrm{s}^{-1}$ and $1.20\,\mathrm{s}^{-1}$, respectively. Correcting for the effects of imperfect spoiling (assuming a diffusivity of $0.8\,\mathrm{\mu m^2/ms}$[11] and a T2 of 250 ms, the latter estimated by assuming that the value of 196 ms at 55 % PVP[11] increases at 44 % PVP due to the higher water concentration, though the result did not depend strongly on this) adjusted these values to $1.25\,\mathrm{s}^{-1}$ and $1.26\,\mathrm{s}^{-1}$, respectively, which are both in good agreement with the estimate of $1.29\,\mathrm{s}^{-1}$ from the inversion recovery experiment. The small remaining discrepancy may be due to small biases in the f_t maps used for the dual flip angle R1 mapping[20] or f_t inhomogeneity over the thick slice used for the inversion recovery experiment.

While there was good agreement between protocols, our estimate of R1 $\sim 1.3\,\mathrm{s}^{-1}$ differed from the literature value[11] of $1\,\mathrm{s}^{-1}$. This discrepancy is probably not just due to temperature, as our measurements were also performed at $\sim 22\,°\mathrm{C}$. Instead, we suspect that this discrepancy is largely explained by the DESPOT1 protocol[21] used to estimate R1 in Pierpaoli et al.[11] being strongly influenced by imperfect spoiling effects due to the choice of RF spoiling increment combined with the long T2 and low diffusivity of the phantom. Simulations using the EPG-X toolbox[8] (assuming the default GE RF spoiling increment of 115.4°,[22] gradient spoiling of 2π dephasing per voxel and the same T2 and diffusivity as above) suggest that a measured R1 $= 1\,\mathrm{s}^{-1}$ would correspond to a real R1 $= 1.19\,\mathrm{s}^{-1}$, which is in better agreement with our estimates.

Figure S21: The spatial distribution of differences between small angle and novel estimator results followed the spatial distribution of the f_t map in the phantom experiment using parameters optimal for estimating R1. f_t was computed using the AFI method. A: R1 and B: A maps estimated using the novel estimators in an exemplary slice. C and D: Relative differences of small angle and novel estimator results show the same spatial pattern as E: the f_t map (interpolated to MPM space). F: Histograms of the f_t-dependence of the errors over the phantom (brighter colour means more voxels in a bin). abs.: absolute value; a.u.: arbitrary units.

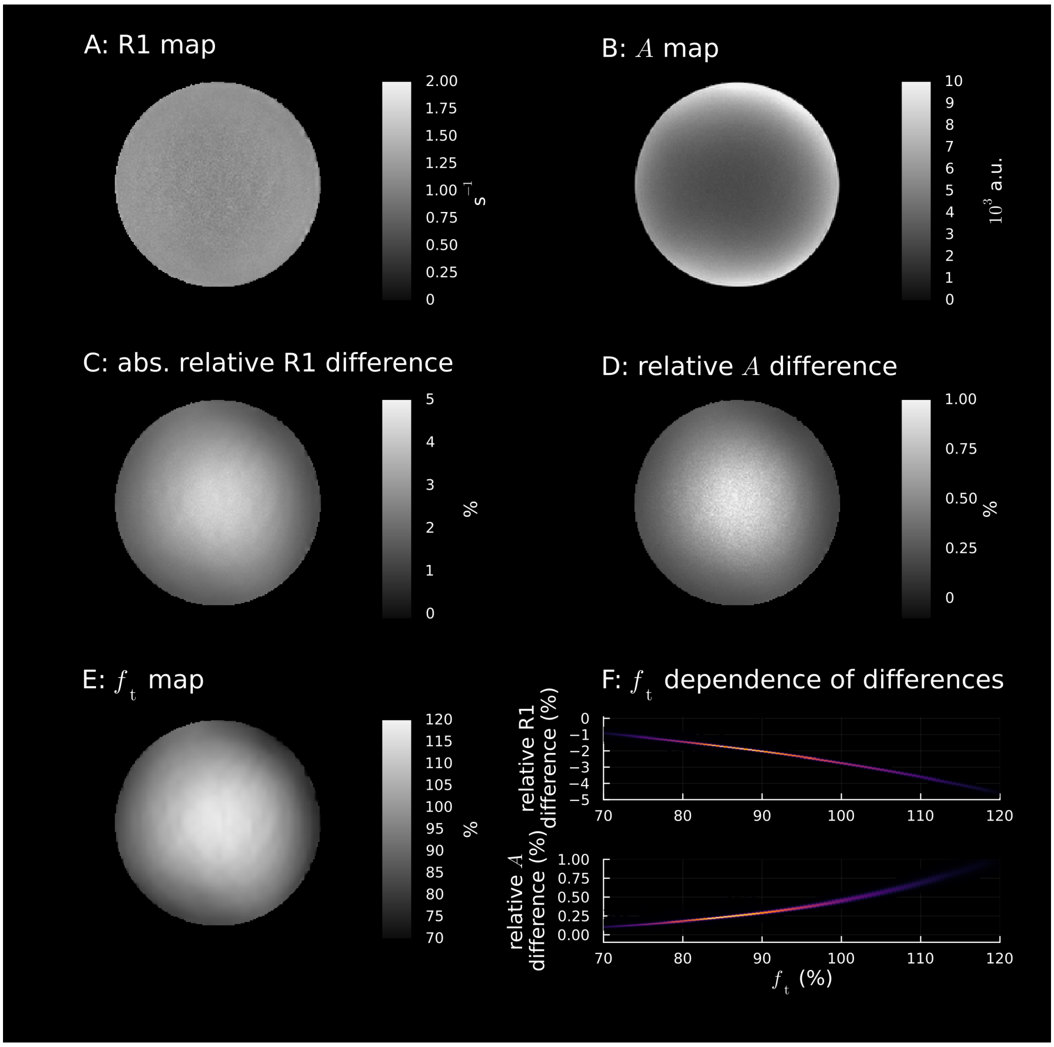

Figure S22: The spatial distribution of differences between small angle and novel estimator results followed the spatial distribution of the f_t map in the phantom experiment using parameters optimal for estimating R1. f_t was computed using the SE/STE method. A: R1 and B: A maps estimated using the novel estimators in an exemplary slice. C and D: Relative differences of small angle and novel estimator results show the same spatial pattern as E: the f_t map (interpolated to MPM space). F: Histograms of the f_t-dependence of the errors over the phantom (brighter colour means more voxels in a bin). abs.: absolute value; a.u.: arbitrary units.

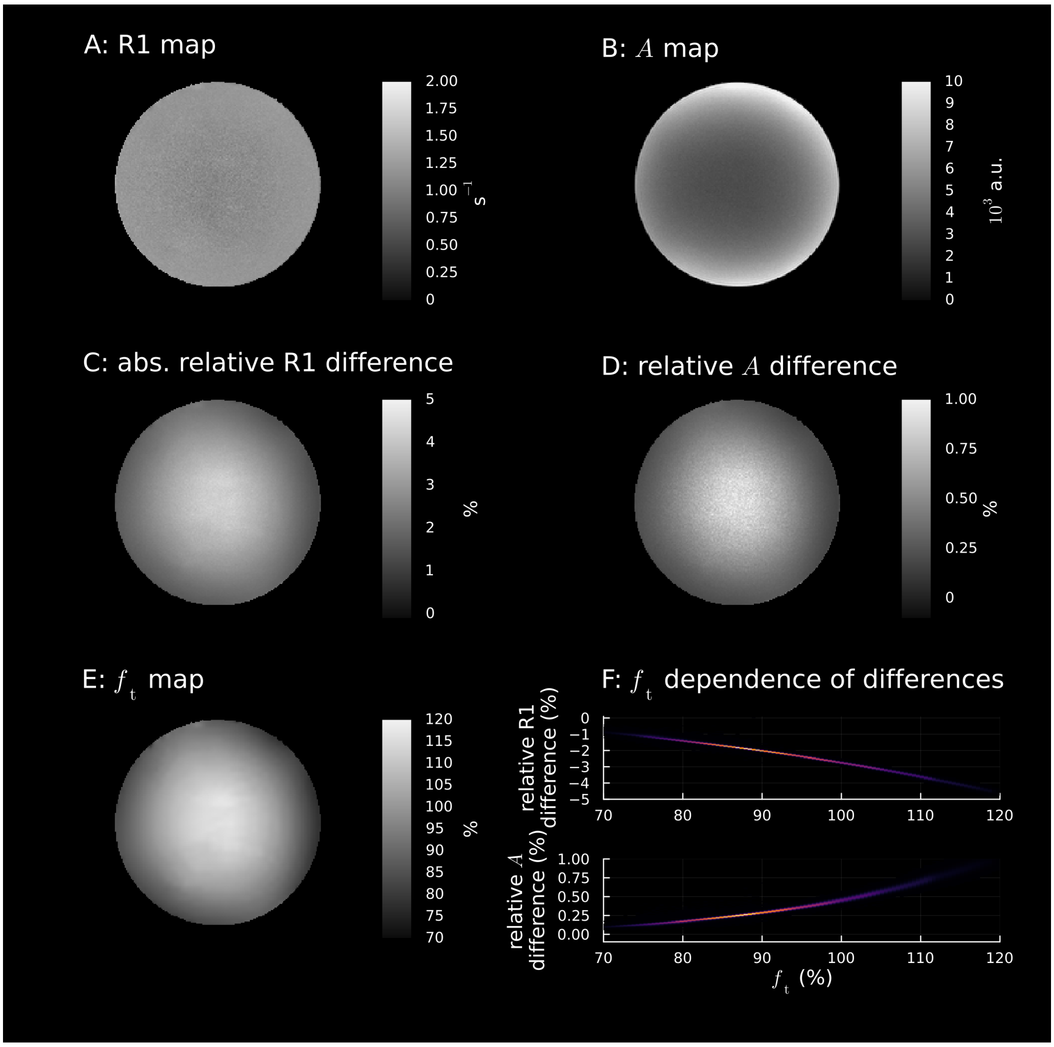

Figure S23: The spatial distribution of differences between small angle and novel estimator results followed the spatial distribution of the f_t map in the phantom experiment using parameters optimal for estimating A. f_t was computed using the AFI method. A: R1 and B: A maps estimated using the novel estimators in an exemplary slice. C and D: Relative differences of small angle and novel estimator results show the same spatial pattern as E: the f_t map (interpolated to MPM space). F: Histograms of the f_t-dependence of the errors over the phantom (brighter colour means more voxels in a bin). abs.: absolute value; a.u.: arbitrary units.

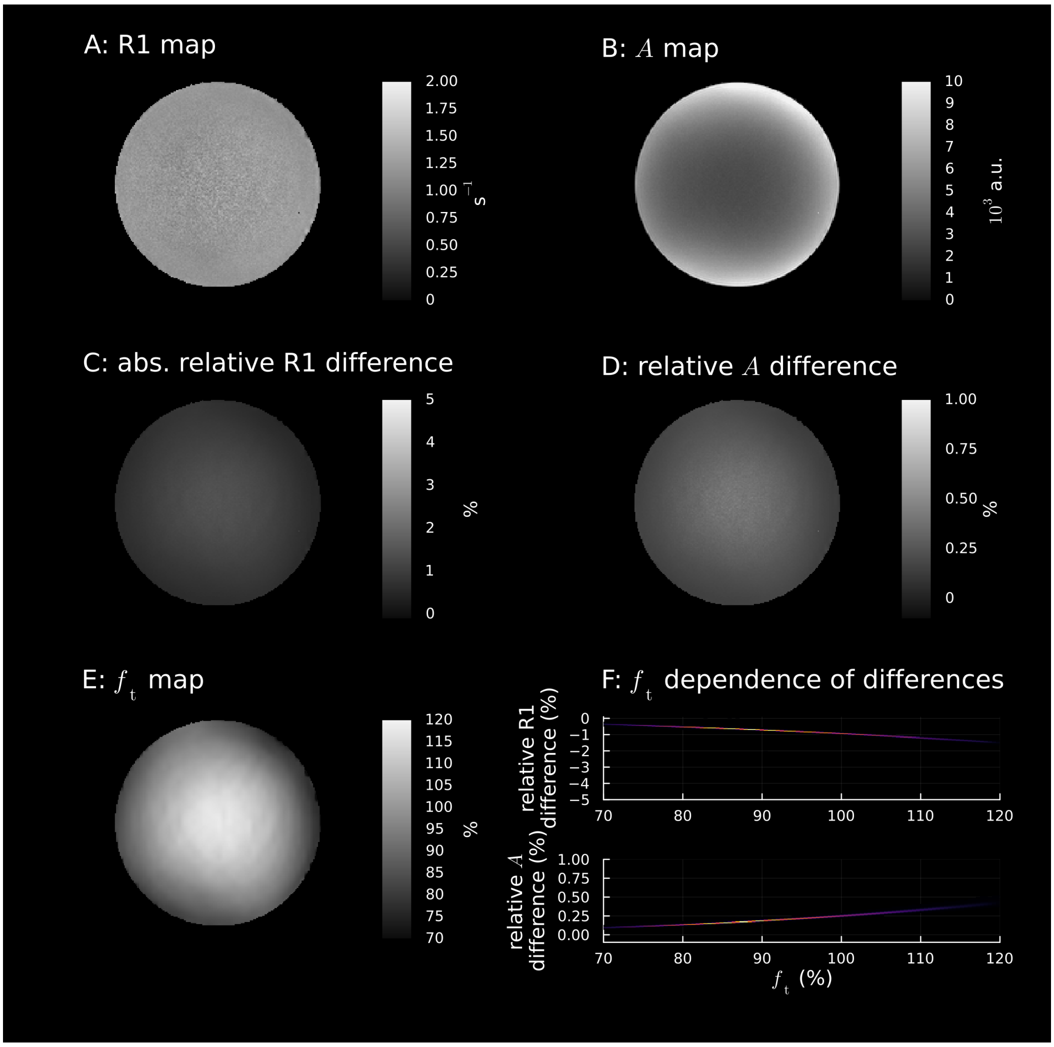

Figure S24: The spatial distribution of differences between small angle and novel estimator results followed the spatial distribution of the f_t map in the phantom experiment using parameters optimal for estimating A. f_t was computed using the SE/STE method. A: R1 and B: A maps estimated using the novel estimators in an exemplary slice. C and D: Relative differences of small angle and novel estimator results show the same spatial pattern as E: the f_t map (interpolated to MPM space). F: Histograms of the f_t-dependence of the errors over the phantom (brighter colour means more voxels in a bin). abs.: absolute value; a.u.: arbitrary units.

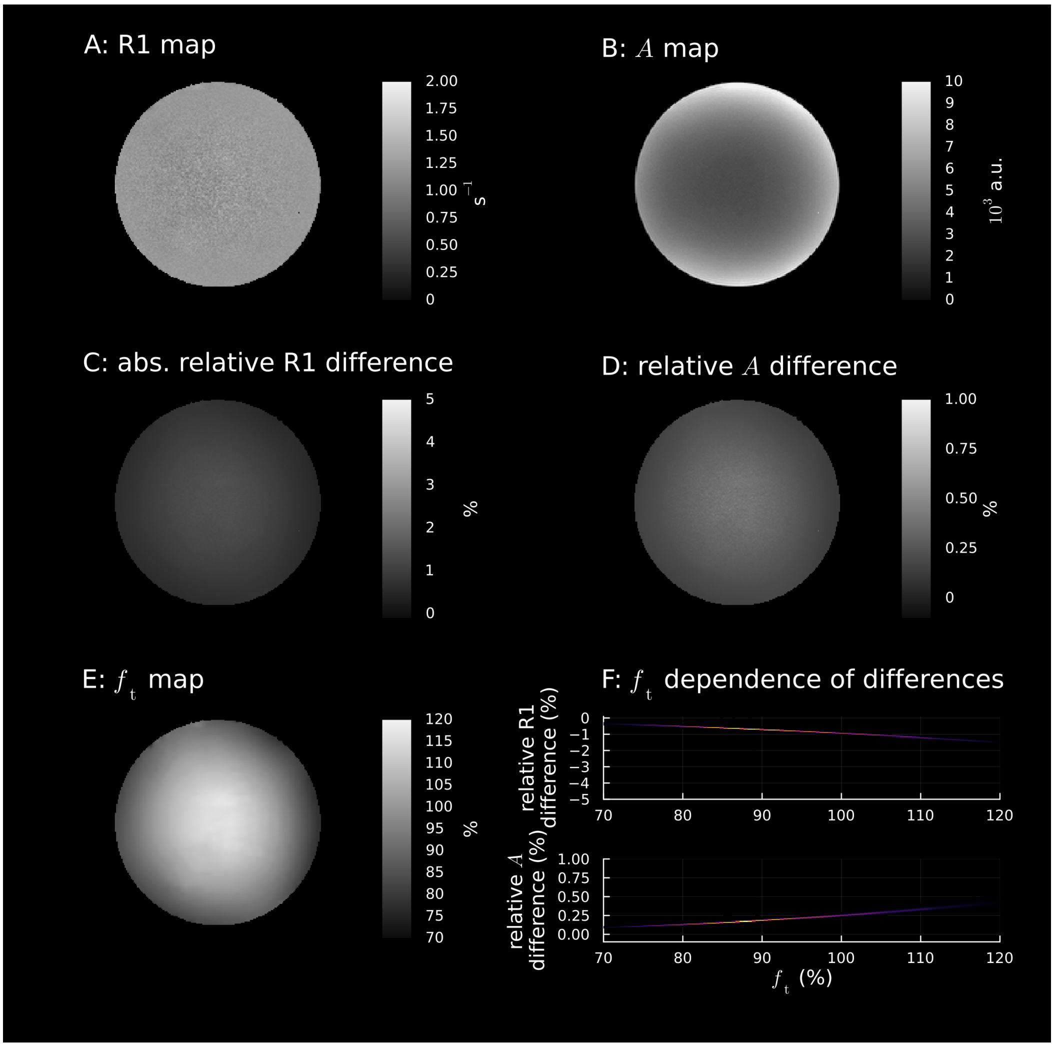

Figure S25: Bland–Altman density plots (brighter colour means more voxels in a bin) showing good agreement between phantom R1 values measured using the two different protocols (upper panel). Correction for imperfect spoiling (see Section S4) removes the small mean difference between the two measurements (lower panel). f_{t} was computed using the AFI method. Blue line: mean difference. Red lines: mean difference ± 1.96(standard deviation of difference).

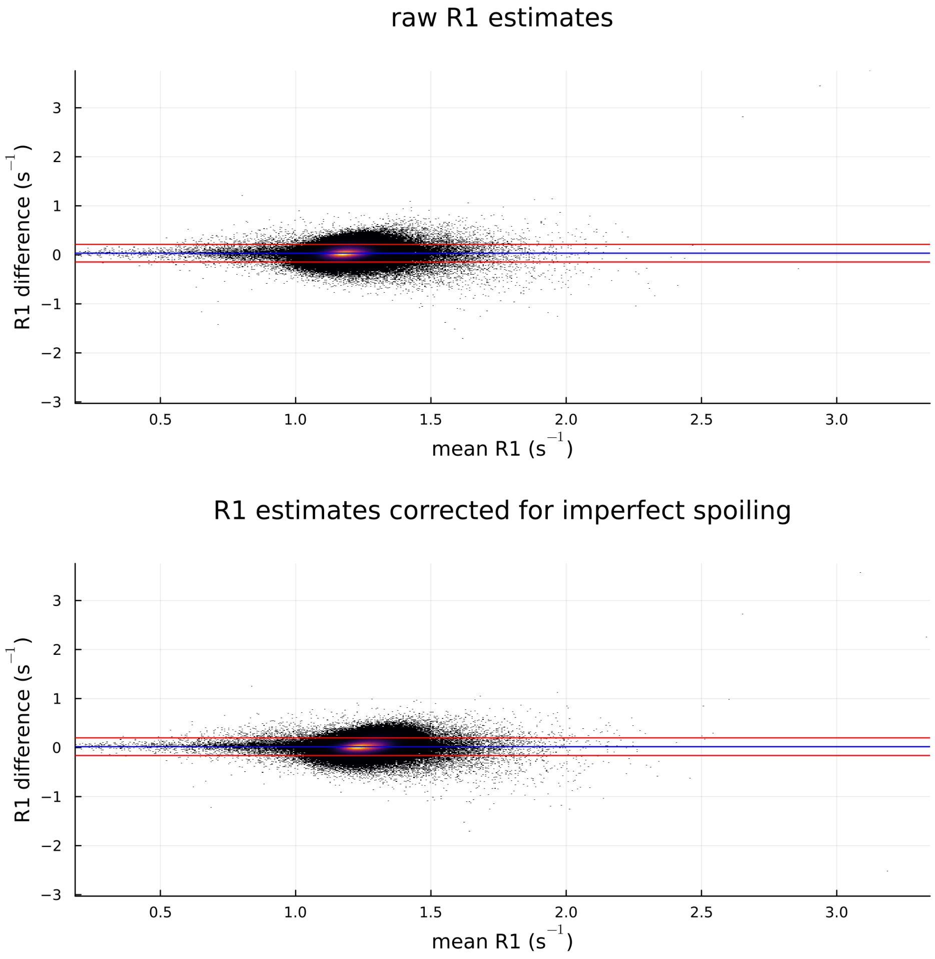

Figure S26: Bland–Altman density plots (brighter colour means more voxels in a bin) showing good agreement between phantom R1 values measured using the two different protocols (upper panel). Correction for imperfect spoiling (see Section S4) removes the small mean difference between the two measurements (lower panel). f_{t} was computed using the SE/STE method. Blue line: mean difference. Red lines: mean difference $\pm$ 1.96(standard deviation of difference).

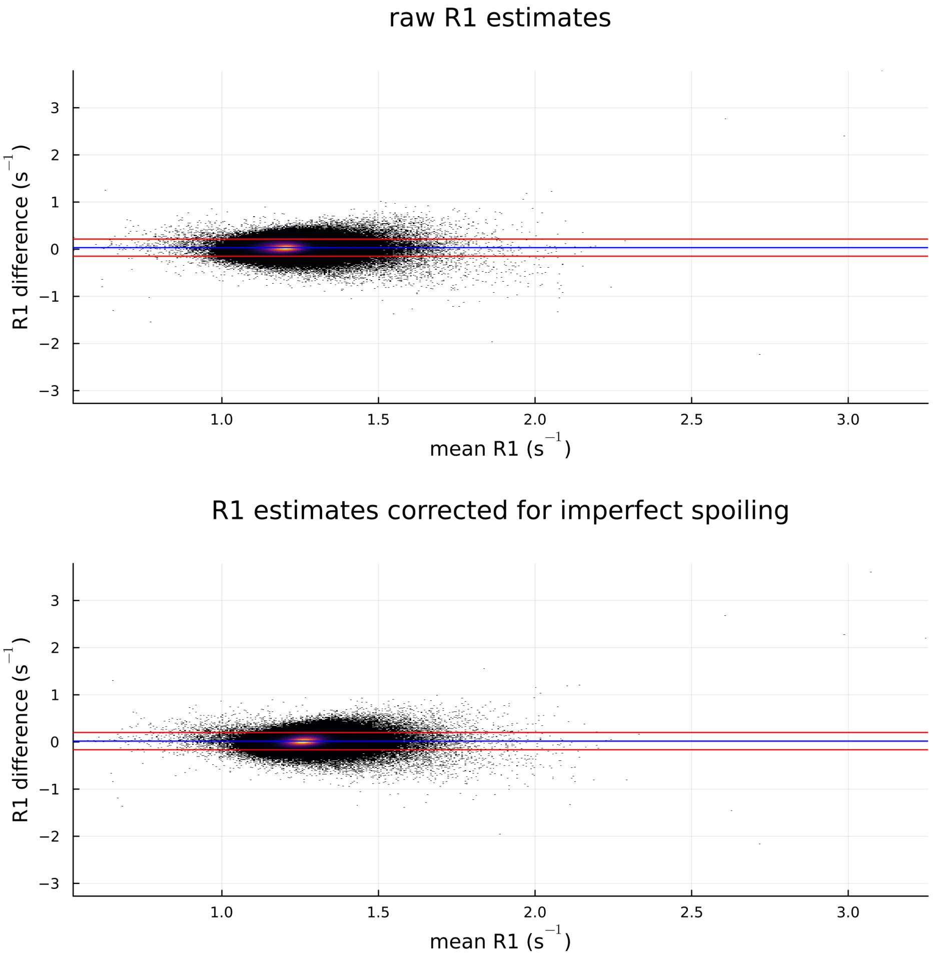

Supplementary References

[1] Dathe H, Helms G. Exact algebraization of the signal equation of spoiled gradient echo MRI. Phys. Med. Biol. 2010;55.15:4231–4245. DOI: 10.1088/0031-9155/55/15/003.

[2] Mohammadi S, Streubel T, Klock L, et al. Error quantification in multi-parameter mapping facilitates robust estimation and enhanced group level sensitivity. Neuroimage. 2022;262:119529. DOI: 10.1016/j.neuroimage.2022.119529.

[3] Rooney WD, Johnson G, Li X, et al. Magnetic field and tissue dependencies of human brain longitudinal ^1H_2O relaxation in vivo. Magn. Reson. Med. 2007;57.2:308–318. DOI: 10.1002/mrm.21122.

[4] Bernstein MA, King KF, Zhou XJ. *Handbook of MRI Pulse Sequences*. Elsevier Science, 2004. DOI: https://doi.org/10.1016/B978-0-12-092861-3.X5000-6.

[5] Helms G, Dathe H, Dechent P. Quantitative FLASH MRI at 3T using a rational approximation of the Ernst equation. Magn. Reson. Med. 2008;59.3. Erratum: Magn. Reson. Med. 63:1136 (2010):667–672. DOI: 10.1002/mrm.21542.

[6] Preibisch C, Deichmann R. Influence of RF spoiling on the stability and accuracy of T1 mapping based on spoiled FLASH with varying flip angles. Magn. Reson. Med. 2009;61.1:125–135. DOI: https://doi.org/10.1002/mrm.21776.

[7] Corbin N, Callaghan MF. Imperfect spoiling in variable flip angle T1 mapping at 7T: Quantifying and minimizing impact. Magn. Reson. Med. 2021;86.2:693–708. DOI: 10.1002/mrm.28720.

[8] Malik SJ, Teixeira RPAG, Hajnal JV. Extended phase graph formalism for systems with magnetization transfer and exchange. Magn. Reson. Med. 2018;80.2:767–779. DOI: 10.1002/mrm.27040.

[9] Eichner C, Paquette M, Müller-Axt C, et al. Detailed mapping of the complex fiber structure and white matter pathways of the chimpanzee brain. Nat. Methods. 2024;21.6:1122–1130. DOI: 10.1038/s41592-024-02270-1.

[10] Eichner C, Anwander A. *Data for "Detailed Mapping of the Complex Fiber Structure and White Matter Pathways of the Chimpanzee Brain" (Nature Methods, https://www.nature.com/articles/s41592-024-02270-1)*. Version V1. Edmond, 2024. DOI: 10.17617/3.O5XSI9.

[11] Pierpaoli C, Sarlls J, Nevo U, Basser PJ, Horkay F. Polyvinylpyrrolidone (PVP) water solutions as isotropic phantoms for diffusion MRI studies. Proc. Intl. Soc. Mag. Reson. Med. 17. 2009:1414. URL: https://archive.ismrm.org/2009/1414.html.

[12] Breuer FA, Blaimer M, Mueller MF, et al. Controlled aliasing in volumetric parallel imaging (2D CAIPIRINHA). Magn. Reson. Med. 2006;55.3:549–556. DOI: 10.1002/mrm.20787.

[13] Lutti A, Hutton C, Finsterbusch J, Helms G, Weiskopf N. Optimization and validation of methods for mapping of the radiofrequency transmit field at 3T. Magn. Reson. Med. 2010;64.1:229–238. DOI: 10.1002/mrm.22421.

[14] Yarnykh VL. Actual flip-angle imaging in the pulsed steady state: A method for rapid three-dimensional mapping of the transmitted radiofrequency field. Magn. Reson. Med. 2007;57.1:192–200. DOI: 10.1002/mrm.21120.

[15] Griswold MA, Jakob PM, Heidemann RM, et al. Generalized autocalibrating partially parallel acquisitions (GRAPPA). Magn. Reson. Med. 2002;47.6:1202–1210. DOI: 10.1002/mrm.10171.

[16] Nehrke K. On the steady-state properties of actual flip angle imaging (AFI). Magn. Reson. Med. 2009;61.1:84–92. DOI: 10.1002/mrm.21592.

[17] Tabelow K, Balteau E, Ashburner J, et al. hMRI – A toolbox for quantitative MRI in neuroscience and clinical research. Neuroimage. 2019;194:191–210. DOI: 10.1016/j.neuroimage.2019.01.029.

[18] Barral JK, Gudmundson E, Stikov N, Etezadi-Amoli M, Stoica P, Nishimura DG. A robust methodology for in vivo T1 mapping. Magn. Reson. Med. 2010;64.4:1057–1067. DOI: 10.1002/mrm.22497.

[19] Karakuzu A, Boudreau M, Duval T, et al. qMRLab: Quantitative MRI analysis, under one umbrella. J. Open Source Softw. 2020;5.53:2343. DOI: 10.21105/joss.02343.

[20] Lee Y, Callaghan MF, Nagy Z. Analysis of the Precision of Variable Flip Angle T1 Mapping with Emphasis on the Noise Propagated from RF Transmit Field Maps. Front. Neurosci. 2017;11 - 2017. DOI: 10.3389/fnins.2017.00106.

[21] Deoni SCL. High-resolution T1 mapping of the brain at 3T with driven equilibrium single pulse observation of T1 with high-speed incorporation of RF field inhomogeneities (DESPOT1-HIFI). J. Magn. Reson. Imaging. 2007;26.4:1106–1111. DOI: 10.1002/jmri.21130.

[22] Wen J, Sukstanskii AL, Yablonskiy DA. Phase-sensitive B1 mapping: Effects of relaxation and RF spoiling. Magn. Reson. Med. 2018;80.1:101–111. DOI: 10.1002/mrm.27009.
